# Trajectories of resting energy expenditure and performance of predictive equations in children hospitalized with an acute illness and malnutrition: a longitudinal study

**DOI:** 10.1038/s41598-024-53791-w

**Published:** 2024-02-13

**Authors:** Farzana Afroze, Farnaz Khoshnevisan, Philliness Prisca Harawa, Zahidul Islam, Celine Bourdon, Stanley Khoswe, Munirul Islam, Shafiqul Alam Sarker, Farhana Islam, Abu Sadat Mohammad Sayeem Bin Shahid, Koen Joosten, Jessie M. Hulst, Chisomo Eneya, Judd L. Walson, James A. Berkley, Isabel Potani, Wieger Voskuijl, Tahmeed Ahmed, Mohammod Jobayer Chisti, Robert H. J. Bandsma

**Affiliations:** 1 The Childhood Acute Illness and Nutrition (CHAIN) Network, Nairobi, Kenya; 2 Nutrition Research Division (NRD), International Centre for Diarrhoeal Disease Research, Bangladesh (icddr,b), Dhaka, Bangladesh; 3 Translational Medicine, Hospital for Sick Children, Toronto, Canada; 4 Department of Paediatrics and Child Health, Kamuzu University of Health Sciences, Blantyre, Malawi; 5 Department of Neonatal and Paediatric Intensive Care, Division of Paediatric Intensive Care, Erasmus MC, Sophia Children’s Hospital, Rotterdam, The Netherlands; 6 Division of Paediatric Gastroenterology, Hepatology and Nutrition, The Hospital for Sick Children, Toronto, Canada; 7 Department of Nutritional Sciences, Faculty of Medicine, University of Toronto, Toronto, Canada; 8 Department of International Health, Bloomberg School of Public Health, Johns Hopkins University, Baltimore, USA; 9 Centre for Tropical Medicine and Global Health, University of Oxford, Oxford, UK; 10 Clinical Research Department, KEMRI–Wellcome Trust Research Programme, Kilifi, Kenya; 11 Centre for Global Child Health, Hospital for Sick Children, Toronto, Canada; 12 Department of Paediatrics, Amsterdam Centre for Global Child Health, Emma Children’s Hospital, Amsterdam UMC, University of Amsterdam, Amsterdam, The Netherlands; 13 Office of Executive Director, International Centre for Diarrhoeal Disease Research, Bangladesh (icddr, b), Dhaka, Bangladesh; 14 Department of Biomedical Sciences, Kamuzu University of Health Sciences, Blantyre, Malawi

**Keywords:** Diseases, Health care, Medical research

## Abstract

There is scarce data on energy expenditure in ill children with different degrees of malnutrition. This study aimed to determine resting energy expenditure (REE) trajectories in hospitalized malnourished children during and after hospitalization. We followed a cohort of children in Bangladesh and Malawi (2–23 months) with: no wasting (NW); moderate wasting (MW), severe wasting (SW), or edematous malnutrition (EM). REE was measured by indirect calorimetry at admission, discharge, 14-and-45-days post-discharge. 125 children (NW, n = 23; MW, n = 29; SW, n = 51; EM, n = 22), median age 9 (IQR 6, 14) months, provided 401 REE measurements. At admission, the REE of children with NW and MW was 67 (95% CI [58, 75]) and 70 (95% CI [63, 76]) kcal/kg/day, respectively, while REE in children with SW was higher, 79 kcal/kg/day (95% CI [74, 84], *p* = 0.018), than NW. REE in these groups was stable over time. In children with EM, REE increased from admission to discharge (65 kcal/kg/day, 95% CI [56, 73]) to 79 (95% CI [72, 86], *p* = 0.0014) and was stable hereafter. Predictive equations underestimated REE in 92% of participants at all time points. Recommended feeding targets during the acute phase of illness in severely malnourished children exceeded REE. Acutely ill malnourished children are at risk of being overfed when implementing current international guidelines.

## Introduction

In resource-limited settings, malnutrition is common in children hospitalized for an acute illness^1^ and is associated with case fatality ratios of 8–25%, despite antibiotic treatment and protocolized feeding^2^. Children commonly enter a downward cycle where illness worsens their malnutrition and vice versa due to enhanced metabolic needs, which negatively impacts clinical outcomes^3^. Acutely ill children with the most severe forms of malnutrition are managed according to guidelines developed by the World Health Organization (WHO)^4,5^. However, the recommended energy intake is based on very limited scientific evidence and may need to be more optimal for these highly vulnerable children. Indirect calorimetry (IC) can accurately measure resting energy expenditure (mREE) in critically ill patients^6^. While it is recommended to calculate feed requirements based on measured energy expenditure^6^, this is rarely done. Barriers in lower-resource settings include cost, equipment, and the need for specific expertise^7^. Therefore, REE is usually predicted with equations (e.g. WHO, Schofield, or Harris-Benedict) based on easily obtained measures (i.e. age, sex, and anthropometry)^8^. However, the accuracy of these equations has been questioned, particularly for use in critically ill malnourished children^9^.

Most studies measuring REE in critically ill children have been conducted in high-resource countries or in the 1960s and 70s using techniques such as early open-circuit systems and cumulative heartbeat counters^10–13^. Except for a small study by Rising et al. REE was measured chiefly in the hospital during the stabilization phase of malnourished children^14^. Thus, their usefulness in extrapolating appropriate feeding targets for their different treatment phases is limited. These phases include: (1) stabilization, when children are routinely provided 80–100 kcal/kg/day at hospital admission; (2) nutritional rehabilitation, using higher caloric feeds (up to 220 kcal/kg/day) with either local or commercial milk or ready-to-use therapeutic food (RUTF)^4,5^. Therefore, there is a clear need to assess REE and caloric demands throughout phases of illness and recovery in hospitalized children with and without malnutrition. The objectives of this study were: (1) to describe longitudinal changes in REE measured by IC in acutely ill hospitalized children with no, moderate and severe wasting and with edematous malnutrition; (2) to examine the clinical and socio-demographic correlates of mREE; (3) to evaluate the accuracy of predictive equations commonly used to calculate caloric demands in malnourished children and (4) to compare mREE to current clinical feeding targets.

## Results

### Participant sociodemographic and clinical characteristics

A total of 149 children were enrolled; 72 (48%) were Malawian, and 77 (52%) were Bangladeshi (Supplementary Fig. S1). From these, 24 participants were excluded: 8 (5%) died before the first indirect calorimetry (IC) measurement, 11 (7%) withdrew consent or left against medical advice, and 5 (3%) had unsuccessful IC at all-time points. The sociodemographic and clinical characteristics of included participants are presented in Table 1. 64% were male, and the median (IQR) age was 9 months (6, 14). Diarrhea, dehydration, and pneumonia were the most common medical diagnoses, and these conditions often co-occurred, with 24 children (21%) having both dehydration and diarrhea, while 15 (13%) had dehydration, diarrhea, and pneumonia (Supplementary Fig. S2). The median (IQR) length of hospital stay was: NW, 3 (2, 3); MW, 4 (3, 5); SW, 4 (3, 7) and EM, 5 (4, 7) days.

**Table 1:** Socio-demographic and clinical characteristics at admission of hospitalized children by nutritional status.

	No wasting	Moderate wasting	Severe wasting	Edematous malnutrition
	n = 23	n = 29	n = 51	n = 22
Demographics				
Site, Bangladesh	7 (30%)	26 (90%)	33 (65%)	8 (36%)
Sex, male	16 (70%)	19 (66%)	33 (65%)	12 (55%)
Age (months)	11 (8, 15)	8 (6, 11)	7 (5, 12)	13 (7, 21)
Reported breastfeeding	20 (87%)	23 (79%)	39 (76%)	7 (32%)
Birth weight (Kg)	3∙1 (3.0, 3.3)	2∙8 (2.5, 3.0)	2∙1 (2.0, 3.0)	2∙7 (2.3, 3.1)
Anthropometry at admission				
Weight (Kg)	8∙2 (7.6,.9.1)	6.6.(6.0, 6.9)	5.1 (4.4, 6.2)	6.9 (6.1, 8.2)
Height (cm)	71 (69, 74)	67 (64, 70)	64 (58, 70)	72 (63, 75)
MUAC (cm)	13.6 (13.1, 14.4)	12.1 (11.9, 12.2)	10.7 (10.2, 11.3)	12.4 (11.2, 13.0)
WLZ	− 0.5 (− 1.1, 0.1)	− 2.0 (− 2.4, − 1.6)	− 3.4 (− 3.8, − 2.6)	− 1.4 (− 2.7, − 0.6)
WAZ	− 1.4 (− 1.9, − 0.2)	− 2.7 (− 3.2, − 1.9)	− 4.1 (− 4.7, − 3.5)	− 2.8 (− 3.6, − 1.7)
LAZ	− 1.8 (− 2.2, − 0.9)	− 1.7 (− 3.0, − 0.7)	− 3.2 (− 4.2, − 2.1)	− 2.9 (− 3.4, − 2.5)
Clinical presentation				
Dehydration	4 (17%)	13 (45%)	21 (41%)	6 (27%)
Pneumonia	7 (30%)	6 (21%)	16 (31%)	8 (36%)
Acute diarrhea	9 (39%)	26 (90%)	40 (78%)	15 (68%)
Sepsis	4 (17%)	1 (3.4%)	7 (14%)	4 (18%)
Malaria, positive	5 (22%)	0 (0%)	1 (2.0%)	0 (0%)
HIV, positive	1 (4.3%)	1 (3.4%)	5 (9.8%)	3 (14%)
Temperature ≥ 38 ºC	10 (43%)	4 (14%)	8 (16%)	3 (14%)
Tachypnea	6 (26%)	3 (10%)	9 (18%)	5 (23%)
Tachycardia	3 (13%)	0 (0%)	8 (16%)	5 (23%)
Blood glucose (mmol/L)	6.5 (5.2, 7.5)	5.7 (5.0, 6.4)	5.7 (4.9, 6.7)	6.0 (4.9, 6.9)
Hemoglobin (g/dl)	10.6 (9.9, 10.9)	10.4 (9.3, 11.2)	10.2 (9.3, 11.3)	10.8 (9.6, 11.4)
WBC (10^9^ cells/L)	16.4 (10.6, 20.5)	13.9 (10.5, 16.3)	13.8 (12.1, 18.4)	15.1 (12.1, 17.4)
Outcome				
Length of hospital stay				
≤ 2 days	10 (43%)	3 (10%)	8 (16%)	2 (9.1%)
3–5 days	11 (48%)	22 (76%)	27 (53%)	11 (50%)
> 5 days	2 (8.7%)	4 (14%)	16 (31%)	9 (41%)
Death	0 (0%)	1* (3.4%)	1 (2.0%)	0 (0%)

Data are presented as median (IQR) or frequency (%).

*WLZ* weight-for-length z*-*score, *WAZ* weight-for-age z*-*score, LAZ length-for-age z*-*score, *MUAC* mid upper arm circumference, *WBC* white blood cells.

*Indicates participant who died after 45-days post-discharge.

### Resting energy expenditure

**Figure 1:**
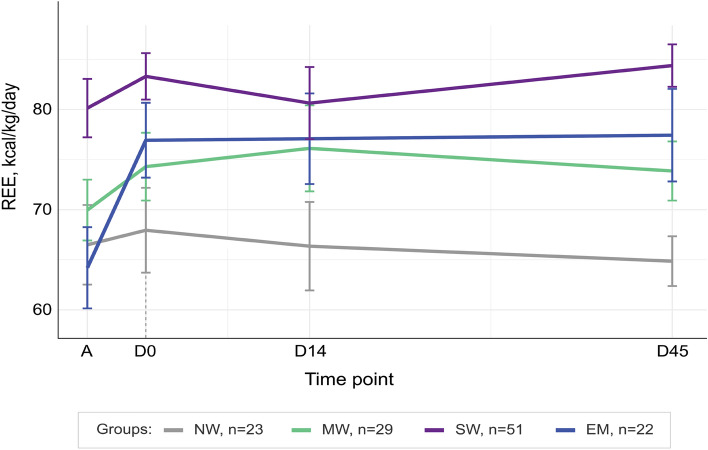
Resting energy expenditure (REE) of children by nutritional status. REE is presented as weight-corrected REE (kcal/kg/day) at admission (A), discharge (D0), 14-days (D14), and 45-days (D45) post-discharge. Lines represent mean group trajectory colored coded as per legend. Error bars indicate standard error of the mean. Vertical dashed line (grey) highlights point of discharge. *NW* no wasting, *MW* moderate wasting, *SW* severe wasting, *EM* edematous malnutrition.

We analyzed a total of 401 REE measurements [Admission (A), n = 95; Discharge (D0), n = 112; Day-14 post discharge (D14), n = 96; and Day-45 post discharge (D45), n = 98] obtained with IC. Based on pre-established quality control cut-offs, 76/477 measures (16%) were removed. The main reasons for exclusion were a respiratory quotient (RQ) > 1.1 (n = 29), RQ < 6.7 (n = 24), high variability of VCO2 or VO2 (n = 17), and fever (n = 6). Supplementary Table S2 details anthropometry, the number of participants with mREE, unadjusted mREE (kcal/kg/day), and RQ values (range, 0.74–0.85) split per nutritional groups and time points. mREE had a strong positive correlation with body weight (Spearman’s rho (399) = 0.62, p < 0.001) and a linked pattern of change across time points (Supplementary Fig. S3). Figure 1 displays the trajectories of mREE (kcal/kg/day) over time by nutritional group (see also Supplementary Table S3). At admission, children with SW had a higher mREE (kcal/kg/day) than those with NW (SW: 79, 95% CI [74, 84] vs. NW: 67, 95% CI [58, 75], showing an increase of 12 kcal/kg/day (95% CI [2.1, 22], p = 0.018). Additionally, children with SW exhibited higher mREE (kcal/kg/day) at admission compared to those with EM (65, 95% CI [56, 73], p = 0.00055). The higher mREE in children with SW was stable over time and did not decrease to D45 post-discharge (Fig. 1 and Table 2). The mREE of children with MW and NW was also stable over time (Fig. 1 and Table 2). However, children with EM showed an increase in mREE (kcal/kg/day) between admission (65, 95% CI [56, 73]) and discharge (79, 95% CI [72, 86]) by 14 kcal/kg/day (95% CI [5.6, 23], p = 0.0014), after which their mREE stabilized in the post-discharge period. The weight of children with EM did not differ between admission and discharge (A, 7.2 kg [95% CI, 6.4, 8.1] versus D0, 7.2 kg [95% CI, 6.3, 8.0], p = 0.35). Also, the mREE pattern remained unchanged if mREE collected at admission was adjusted for either child weight at admission or discharge (Supplementary Fig. S4). By D45, the mREE (kcal/kg/day) of children with EM was higher than those with NW (EM: 79, 95% CI [71, 86] vs. NW: 66, 95% CI [58, 73]), showing a difference of 13 kcal/kg/day (95% CI [2.4, 24], p = 0.017). As expected, mREE per kg decreased with increasing age (Supplementary Table S3).

**Table 2:** Marginal mean estimates of resting energy expenditure (kcal/kg/day) in children hospitalized with acute illness and differing nutritional status.

	Admission (A)	Discharge (D0)	D14	D45	Difference A vs D0		Difference D0 vs D45	
					est. [95% CI]	p	est. [95% CI]	p
*No wasting	66.7 [58.1 − 75.3]	68.8 [62.2 − 75.5]	67.8 [62.1 − 73.5]	65.5 [58.0 − 72.9]	− 2.1 [− 11 − 6.9]	0.65	3.4 [− 5.1 − 11]	0.43
Moderate wasting	69.6 [63.1 − 76.1]	74.5 [68.5 − 80.5]	74.3 [69.1 − 79.4]	73.7 [66.7 − 80.6]	− 4.9 [− 12 − 2.2]	0.18	− 0.84 [− 7.1 − 8.8]	0.83
Severe wasting	78.9 [73.8 − 84.1]	81.8 [77.3 − 86.3]	82.4 [78.5 − 86.2]	83.6 [78.6 − 88.7]	− 2.9 [− 8.4 − 2.7]	0.31	− 1.8 [− 7.6 − 3.9]	0.53
Edematous malnutrition	64.8 [56.4 − 73.2]	79.2 [72.4 − 85.9]	79.0 [73.1 − 84.8]	78.6 [70.7 − 86.4]	− 14.3 [− 23 − 5.6]	0.0014	0.59 [− 8.2 − 9.3]	0.89

Marginal mean estimates and 95% confidence intervals (CI) per group and time point derived from piecewise mixed models fitted with a single knot at discharge and random intercepts per participant. Included predictors: time, nutritional group, time x group interaction, and age at admission. Time was coded as weeks since admission binned within time points of admission (A), discharge (D0), 14 days (D14), and 45 days (D45) post-discharge. Final models were fit using restricted maximum likelihood and full results tables are presented in the Supplementary Tables S4, S5, S6.

*Children with NW- no wasting is the reference group.

### Associations with mREE trajectories

**Figure 2:**
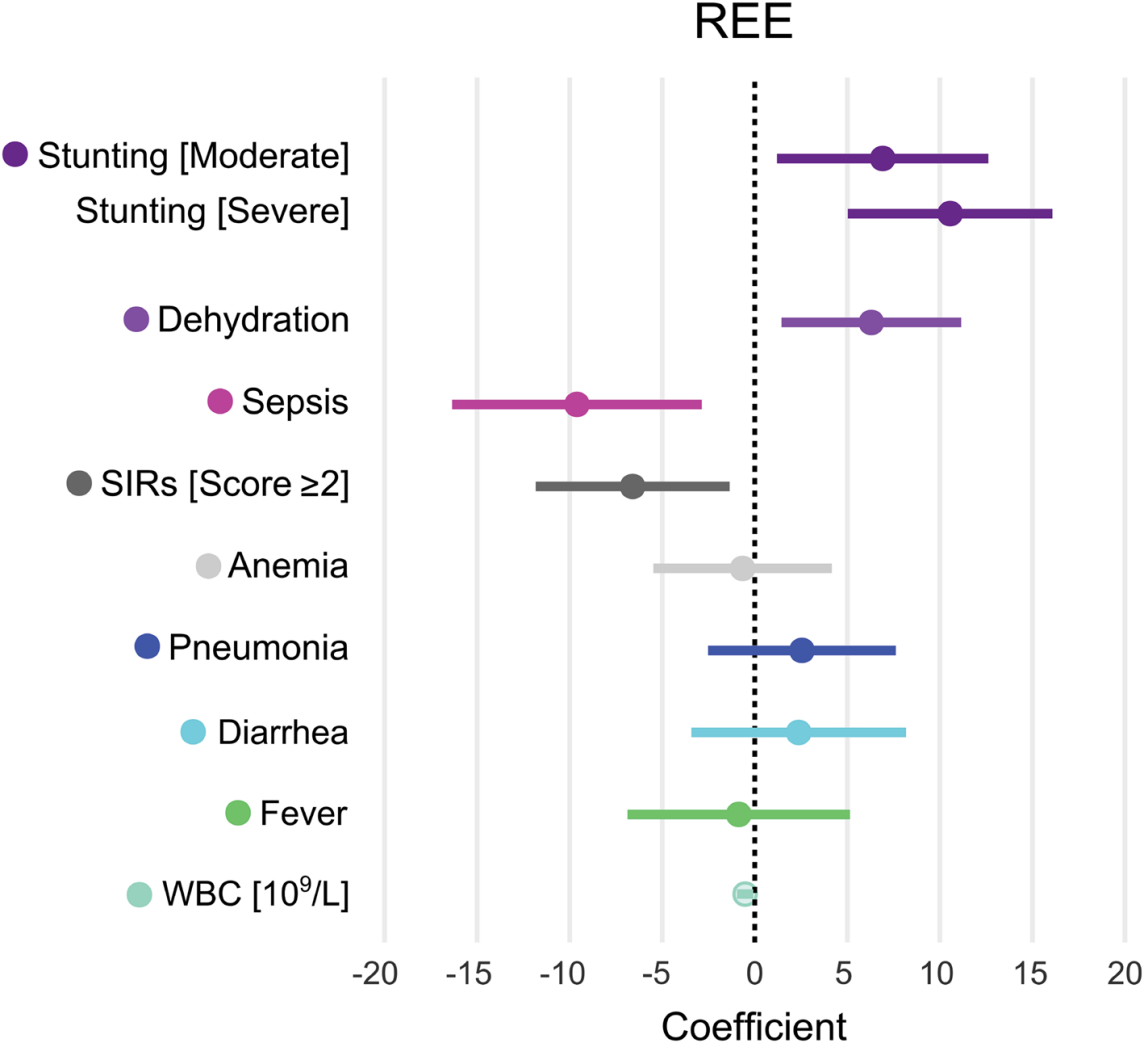
Association between resting energy expenditure and clinical predictors in hospitalized children with varying nutritional status. Regression coefficients derived from piecewise mixed models testing the association between resting energy expenditure and different clinical diagnoses in children hospitalized with acute illness and of varying nutritional status. All models include the `Base` model (i.e. M_0_: REE ~ time × group + age) with an additional clinical variable as per legend. Piecewise models were fitted with a single knot at discharge and with random intercepts per participant. The confidence intervals of significant variables do not cross zero (i.e. dashed black center line). Full results for the nine models are presented in Supplementary Tables S4, S5 and S6.

Figure 2 and Table 3 (Supplementary Table S4, S5, and S6) displays the association between REE and clinical predictors, including stunting, dehydration, sepsis, high SIRS score (i.e.  ≥ 2), anemia, pneumonia, diarrhea, fever, and WBC in children hospitalized with acute illness. Of these, severely stunted children had higher mREE by 11 kcal/kg/day (95% CI [5.0, 16], p < 0.001) and moderately stunted children by 6.9 kcal/kg/day (95% CI [1.2, 13], p = 0.018). The higher mREE associated with SW was not statistically significant when accounting for stunting (Supplementary Table S4). While the variance-inflation factors were not > 5, stunting and wasting were related, with a greater proportion of children with SW and EM being stunted (77% in both groups) compared to 35% in children with NW and 41% with MW (age-adjusted OR: SW vs. NW, 7.5 (95% CI [2.5, 24]); EM vs. NW, 5.9 (95% CI [1.6, 24])). Sepsis was negatively associated with mREE: − 10 kcal/kg/day (95% CI [− 16, − 2.9], p = 0.0051). Dehydration was associated with increased mREE by 6.3 kcal/kg/day (95% CI [1.5, 11]; p = 0.011), and no statistically significant relationships were detected with anemia, pneumonia, diarrhea, or fever. Associations did not change when further adjusting for sex, site, and duration of hospital stay (Supplementary Fig. S5).

**Table 3:** Association between clinical predictors and resting energy expenditure (kcal/kg/day) measured in children hospitalized with acute illness and of differing nutritional status.

	Est. [95% CI]	*p*
Intercept [NW]	72 [62, 82]	< 0.001
Slope during admission [NW]	2∙6 [− 10, 15]	0.68
Change of slope during admission vs post − discharge [NW]	− 3∙1 [− 16, 10]	0.64
Difference in intercept [MW − NW]	− 2∙3 [− 13, 8∙0]	0.66
Difference in intercept [SW − NW]	4∙7 [− 5.2, 15]	0.35
Difference in intercept [EM − NW]	− 7∙7 [− 19, 3∙8]	0.19
Difference in slope during admission [MW − NW]	4∙7 [− 11, 21]	0.57
Difference in slope during admission [SW − NW]	2 [− 123, 17]	0.79
Difference in slope during admission [EM − NW]	18 [0.30, 35]	0.046
Difference in change of slope during admission vs post-discharge [MW − NW]	− 4.3 [− 21, 13]	0.61
Difference in change of slope during admission vs post-discharge [SW − NW]	− 1.3 [− 17, 14]	0.87
Difference in change of slope during admission vs post-discharge [EM − NW]	− 17 [− 36, 0.93]	0.063
Age, months	− 0∙66 [− 1.1, − 0.27]	0.001
Stunting [moderate]	6∙8 [1.4, 12]	0.013
Stunting [severe]	12 [6.7, 17]	< 0.001
Dehydration	5∙4 [1.0, 9.8]	0.016
Sepsis	− 10 [− 17, − 4.2]	0.001
Random effects		
σ^2^	186	
τ_00participant_	66	
ICC	0.26	
N_participants_	125	
Observations	401	
Marginal R^2^/conditional R^2^	0.26/0.46	

Piecewise mixed models were fitted with a single knot at discharge, and with random intercepts per participant allowing to evaluate intercepts, slopes, and differences in slopes between groups during admission versus the post-discharge period. Time was coded as weeks since admission binned within time points. Final models were fit using restricted maximum likelihood. Children with *NW* no wasting (reference group), *MW* moderate wasting, *SW* severe wasting, or *EM* edematous malnutrition.

### Accuracy of the REE predictive equations in malnourished children

**Figure 3:**
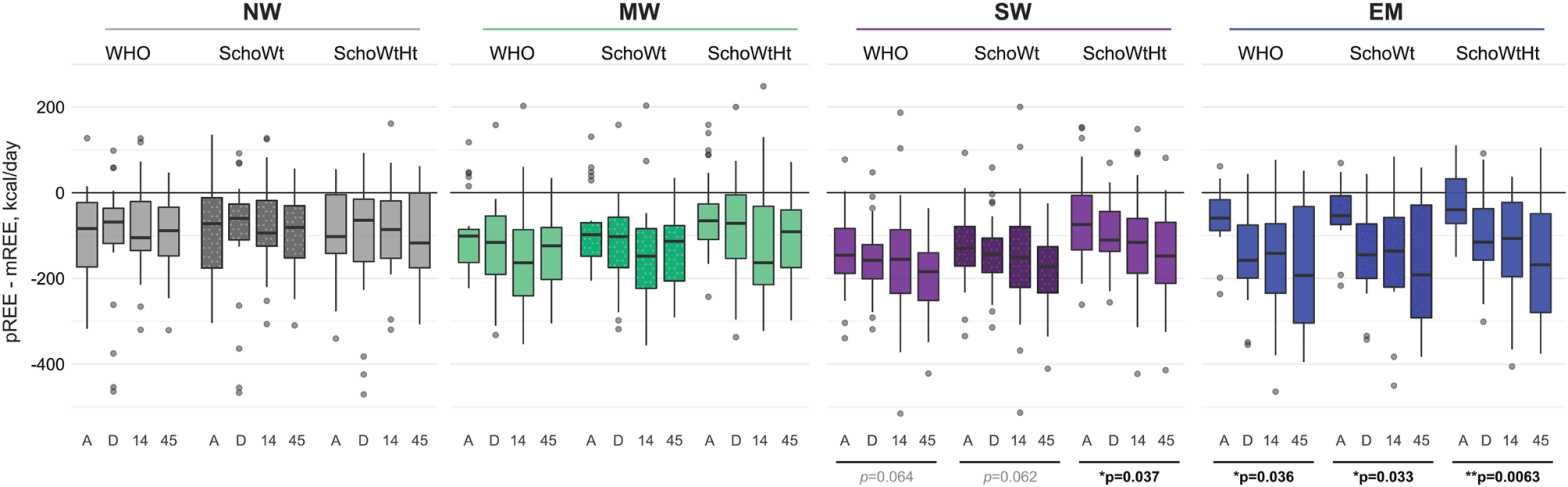
Difference between predicted and measured resting energy expenditure (pREE–mREE). mREE was measured by indirect calorimetry. pREE was estimated by WHO, Schofield with weight correction (Scho-Wt) and Schofield with both weight and height correction (Scho-WtHt) equations. Boxplots for each nutritional group (*NW* no wasting, grey; *MW* moderate wasting, green; *SW* severe wasting, purple, *EM* edematous malnutrition blue) present median and IQR for each equation split by timepoint (A, admission; D, discharge; 14, 14-days post-discharge; 45, 45-days post discharge). Dots beyond whiskers are more than ± 1.5 IQR from the median. Underscore-lines (black) with p-values indicate biases that tend to increase over time (significance: *p < 0.05; **p < 0.01).

**Figure 4:**
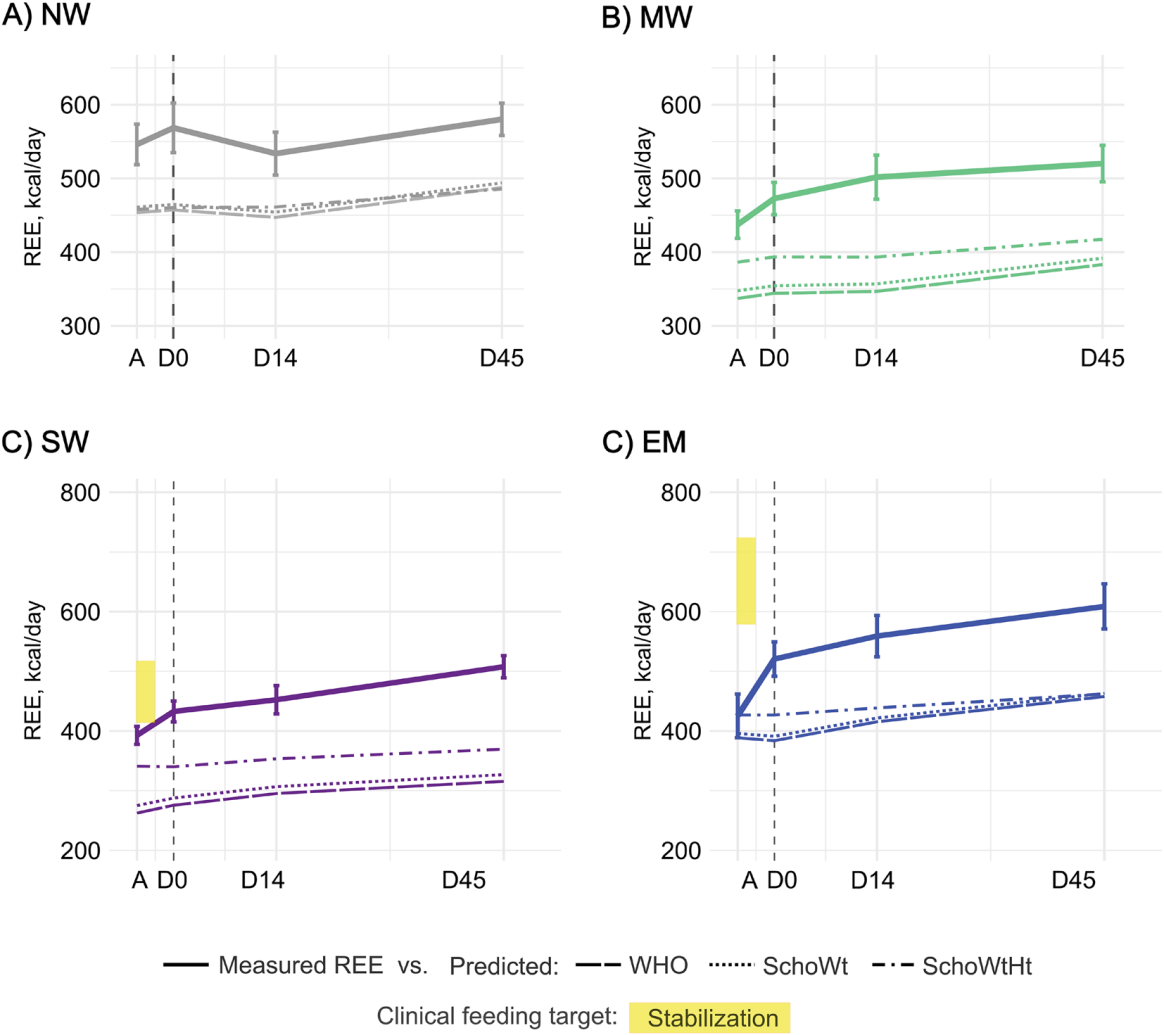
Resting energy expenditure and clinical feeding targets of children by nutritional status and time points. Resting energy expenditure (kcal\day, weight-uncorrected) and clinical feeding targets of children by nutritional status at admission (A), discharge (D0), 14-days (D14), and 45-days (D45) post-discharge. Feeding target range for clinical stabilization (80–100 kcal/kg/day) of severely malnourished children (SW and EM) is indicated by the yellow colored area. Solid lines represent mean group trajectory of measured REE (NW, grey; MW, green; SW, purple; EM, blue). Dashed lines present mean trajectory of predicted REE for each equation as per legend (i.e. WHO, long dash; Schofield with weight correction (SchoWt), dotted; and Schofield with both weight and height correction (SchoWtHt), dot-dashed line). Whiskers indicate standard error of the mean; vertical dashed line (grey), point of discharge. *NW* no wasting, *MW* moderate wasting, *SW* severe wasting, *EM* edematous malnutrition.

All three predictive equations underestimated REE (kcal/day) of hospitalized acutely ill children by an overall average of 129 kcal/day (95% CI [143, 115]) for Schofield weight (Scho-Wt) and 98 kcal/day (95% CI [113, 83]) for Schofield weight and height (Scho-WtHt) (Figs. 3 and 4). The predictive equations without height correction most often underestimated REE and were below the accuracy threshold in 94% of participants, while the frequency of underestimation by the Scho-WtHt equation was 89% (p = 0.0042). Supplementary Table S7 presents the median (IQR) of measured and predicted REE (kcal/day) for each equation by group and time-point and tallies the average percent bias and frequency of children being under or over-estimated. With non-height adjusted equations, the discrepancy between predicted and measured REE was higher in children with SW compared to NW (i.e. WHO equation, NW − 98 kcal/day (95% CI [− 131, − 66]) vs. SW − 164 kcal/day (95% CI [− 185, − 142], p = 0.0061); Scho-Wt equation, NW − 91 kcal/day (95% CI [− 123, − 59]) vs. SW − 152 kcal/day (95% CI [− 173, − 131], p = 0.012). With the Scho-WtHt equation, the discrepancy between predicted and measured REE did not differ between groups; thus, height correction may be valuable for populations with a high prevalence of stunting. However, the discrepancy in severely malnourished children (SW and EM) tended to increase over time, and this pattern was seen for the 3 equations in the EM group and for the SchoWtHt equation in the SW group (Fig. 3). Supplementary Fig. S6 presents Bland–Altman plots comparing measured and predicted REE for each equation. Supplementary Table S8 displays the results of the sensitivity analysis conducted to assess whether Diet induced thermogenesis (DIT) contributed to the differences observed between mREE and pREE. The analysis indicated significant differences between these two methods at all time points, except for the Scho-WtHt equation. The pREE calculated using the Scho-WtHt equation showed comparable results to mREE during admission (p = 0.104).

### Current feeding targets used during stabilization exceed measured metabolic demand of acutely ill children with severe malnutrition

While predictive equations underestimated REE of children hospitalized with acute illness, the currently recommended feeding targets for ill children with severe malnutrition commonly exceeded the measured REE (Fig. 4). *During clinical stabilization*, the feeding target is 80–100 kcal/kg/day, which at admission translated to a mean range between 414 kcal/day (95% CI [382, 446]) and 518 kcal/day (95% CI [485, 550]) in children with SW, and between 579 (95% CI [504, 654]) and 724 kcal/day (95% CI [649, 799]) in children with EM (indicated by yellow area on Fig. 4). The lower feeding target (80 kcal/kg/day) exceeded measured REE at admission in children with EM by 118 kcal/day (95% CI [67, 170], p < 0.0001), while the upper target exceeded measured REE in both SW and EM (excess: SW, by 114 kcal/day (95% CI [84, 143], p < 0.0001); EM, by 263 kcal/day (95% CI [212, 315], p < 0.0001)).

*During rehabilitation*, the feeding target is 180–220 kcal/kg/day, which translated into ranges between 909 kcal/day (95% CI [850, 966]) and 1331 kcal/day (95% CI [1273, 1389]) in children with SW, and between 1256 (95% CI [1177, 1336]) and 1843 kcal/day (95% CI [1733, 1952]) in children with EM. The lower feeding target for rehabilitation (180 kcal/kg/day) exceeded measured REE at discharge in children with SW by 467 kcal/day (95% CI [414, 520], p < 0.0001) and by 708 kcal/day (95% CI [576, 840], p < 0.0001) in children with EM.

## Discussion

This prospective longitudinal study detailed the trajectory of energy expenditure in acutely ill children with varying nutritional status during their hospitalization and in the first six weeks post-discharge. The study demonstrated that (1) children with severe wasting had higher REE (kcal/kg/day) than non-wasted children and that this may be accounted for by stunting; (2) children with edematous malnutrition markedly increased their REE between admission and discharge, a change unlikely to be driven solely by loss of edema; (3) predictive equations systematically underestimated REE and showed large biases beyond the accepted 10% accuracy threshold; (4) energy intake recommended by WHO during the early stages of critical illness exceeds the basic energy requirements of severely malnourished children, more so in EM. To our knowledge, this is the first study exploring the trajectories of REE in sick children across a range of nutritional status, including children with severe malnutrition.

Our work shows that WHO-recommended calories during acute illness in malnourished children (80 to 100 kcal/kg/day) provide energy beyond their basic metabolic requirements^5,15^. The finding is relevant as evidence from the last ten years has demonstrated that overfeeding critically ill children can worsen clinical outcomes^16,17^. During severe infection, catabolism is thought to benefit immunological and metabolic function^18^. Interfering with this catabolic response by overfeeding may negatively impact the ability to effectively combat infections, possibly by disrupting autophagy^19^. Autophagy, a process known to maintain cellular metabolic health, is now recognized as a critical immune meditator of pathogen clearance, a mechanism at the forefront of innate defenses during infection^20^. A large multicenter clinical trial revealed that delaying parenteral nutrition by 1 week and thereby reducing overall caloric intake improved clinical outcomes, including lowering risk of new-onset infections^18,21^. A recent European guideline stated that overfeeding should be avoided in critically ill children^22^. This means that in the acute phase, caloric targets should remain below resting energy expenditure^23^. Therefore, given our data, the high target range currently recommended by WHO could lead to overfeeding of hospitalized children with severe malnutrition, and these should be revised also considering the common practice of assisted feeding by nasogastric tube in this population.

The lower REE at admission in ill children with edematous malnutrition illustrates that these children may present with distinctly altered metabolism compared to children with severe wasting^24,25^. To capture variation possibly linked to edema, we calculated admission REE (kcal/kg/day) using weights collected at admission or discharge or their lowest weight recorded as inpatients. The shift in REE (kcal/kg/day) was negligible. While we cannot rule out the role of fluid accumulation, an unrecognized mechanism likely underlies the low REE at admission in these children, and this result reflects fundamental metabolic differences. Reduced REE in children with EM, specifically compared to healthy controls, has been reported in two small historical studies but without statistical testing^24,26^. Thus, adapting feeding targets for this edematous group might improve their outcome. We found stunting to relate to higher REE seen in children with SW and EM in their post-discharge period, with the highest stunting prevalence. Similar to Pontzer et al.^27^ we found that weight-corrected REE decreased with increasing age. Our study also confirms findings in young infants that markers of sepsis and inflammation are negatively associated with REE^28^. Conversely, we found that dehydration was significantly associated with increased REE. Dehydration with acidosis is expected to raise metabolic needs with increased respiratory rate.

Although equations to predict energy requirements have been validated in children^29^, they have not been evaluated in sick, malnourished children. We found predictive equations to systematically underestimate their actual energy expenditure. Several recent studies found predictive equations to be inaccurate in critically sick children^29^ and in children with failure to thrive^29,30^. Our post-discharge data also suggests that the bias of predictive equations increase over time in malnourished children recovering from an illness that could be linked to an augmented anabolic state and catch-up growth.

This study has several limitations. We used actual body weight for calculating the weight-corrected REE. However, to mitigate potential bias, the lowest edema-free weight recorded during the hospital stay, with weight recorded daily, was employed. Although, adjusted body weight, accounting for the difference between actual body weight and ideal body weight (IBW), has been suggested for edematous children, there is little consensus on the best method for calculating IBW^31^. As discussed above, our study did not allow us to determine precisely the effect of fluid accumulation in children with EM. It is also important to realize that some children with SW could have had some fluid accumulation that was not clinically apparent, influencing their REE results Performing IC was challenging in this population, some children either moved too much or showed signs of irritability or cried during the procedure, leading to exclusion of those IC assessments. We determined it was clinically not safe to sedate children to be able to complete IC assessments during a phase of critical illness. Our study could not perform IC in a completely fasted state, given the risk of hypoglycemia in severely malnourished children. The repeated IC measurements covering the whole length of acute and rehabilitation phase in children with different categories of nutritional status is the main strength of the study.

We did not estimate the total energy expenditure (TEE), which comprises three elements, namely, basal energy expenditure (BEE, ~ 60–75% of TEE), physical activity-related energy expenditure (~ 15–30% of TEE), and dietary thermogenesis (DIT, ~ 10% of TEE). Thus, the REE, which is the combination of BEE and DIT, is usually lower than TEE^32^. Therefore, the WHO-recommended energy estimation is not the same as the measured REE in this study. We did not assess physical activity (PA), which is highly variable. In hospitalized patients with illness, REE may mirror TEE due to minimal physical activity^3,33^. However, during the outpatient phase when children return to their home environment, TEE increases due to heightened physical activity. Several studies suggest that the mean PA of recovering SAM children is generally low, with no significant difference between boys and girls. Age exhibits a negative correlation with PA, while PA significantly increases with rising WHZ and MUAC. In moderately malnourished children, PA exceeds that of SAM children by more than two times^34,35^. Therefore, it is crucial to consider the levels of PA when determining the energy requirements of malnourished children in outpatient care. In order to determine energy requirements, one should also take into account, a potential disease factor not fully captured by the REE, a growth factor and the energy absorption coefficient as also recommended in the latest WHO guideline on wasting^36^.

## Conclusions

In conclusion, this study suggests that energy requirements differ among children with acute illness and varying nutritional status up to 45 days post-discharge and cannot be adequately determined using existing predictive equations. Current feeding targets in acutely ill children with severe malnutrition often exceed their metabolic demands. Applying current management guidelines could lead to overfeeding these children, especially those with edematous malnutrition. Our results highlight the urgent need for randomized clinical trials to determine the optimal energy and protein requirements for managing acutely ill, severely malnourished children. Also, our study provides a strong foundation for more precisely estimating the energy requirements of children managed for malnutrition as outpatients after recovering from critical illness. Our work can inform WHO and others in their effort to base the nutritional management of ill malnourished children on evidence.

## Methods

### Study design and population

From September 2018 to April 2020, we conducted a prospective longitudinal study in Bangladesh and Malawi to determine REE in hospitalized critically ill children. Our study used the infrastructure of the Childhood Acute Illness & Nutrition (CHAIN) Network, a large multinational cohort study that aims to optimize the management of sick and malnourished children treated in resource-limited contexts to enhance childhood survival and growth^37^. Prior to starting, hospitals were audited for capacity and methodologies were harmonised through extensive cross-site trainings^38^. For this sub-study, we included children aged 2 to 23 months, admitted to hospital for acute illness and residing within the catchment areas of either Dhaka Hospital of the International Centre for Diarrhoeal Disease Research (icddr,b), Dhaka, Bangladesh, or Queen Elizabeth Central Hospital (QECH), Blantyre, Malawi as detailed in the CHAIN protocol^37^. Written informed consent was obtained from each child's parent or legal guardian before enrollment. Exclusion criteria were: inability to tolerate oral feeds in his/her usual state of health, a terminal illness, known chromosomal abnormality, cerebral palsy, admission for surgery, trauma, or a condition likely to require surgery within 6 months. All children received care as per WHO inpatient guidelines, including for the management of SM^5,15^. Malaria and HIV rapid diagnostic tests were performed with appropriate pre-and post-test counseling.

### Screening and group classification

As per CHAIN protocol, children were screened and recruited in strata based on mid-upper arm circumference (MUAC)^37^. Each site designated a specific day to commence weekly recruitment. The first eligible child in each stratum was identified and approached for consent. While screening continued throughout the week, enrollment ceased once the weekly target of 3–5 children was achieved. At admission and at each time point after that, children were also weighed (Seca model 354, Hamburg, Germany), and their lengths were measured by two independent observers using a length board (Seca 416). The process was repeated if the inter-observer discrepancy was more than 7mm. Weight-for-length (WLZ), weight-for-age (WAZ), and length-for-age (LAZ) Z scores were calculated using WHO Anthro and macros software (ver. 3.2.2, WHO)^39^ For this sub-study children ≥ 6 months of age were classified into four nutritional groups based on WHO criteria: (1) no wasting (NW, WLZ > − 2 standard deviations (SD) and MUAC > 12.5cm); (2) moderate wasting (MW, WLZ between ≤ − 2 and ≥ − 3 SD or MUAC between 11.5 and 12.5cm); (3) severe wasting (SW, WLZ < − 3 SD or MUAC < 11.5 cm) and (4) edematous malnutrition (EM, presence of bilateral pitting edema involving both feet)^15^. For children aged < 6 months, the MUAC cut offs were lowered by 0.5cm^37^.

### Management of severely wasted and edematous children

Severely malnourished children were managed according to the WHO guidelines and the national guidelines of Malawi and Bangladesh during both the inpatient and outpatient phases^4,40^. In the initial stabilization phase, they received F-75 or an equivalent diet (Milk suji) to meet feeding targets of 80–100 kcal/kg/day. Once children were ready to enter the rehabilitation phase, characterized by recovery from acute illness, the return of appetite, and the loss of edema fluid, they were discharged from their respective wards and transferred to an observational ward for four days. During the rehabilitation phase, children were gradually transitioned to achieve a calorie intake of 180–220 kcal/kg/day. In Malawi, the Ready-to-Use Therapeutic Food (RUTF) contained 500 kcal per 92 g, while in Bangladesh, locally prepared nutritious foods (milk suji-100, Khichuri, a porridge made with rice, lentils, vegetables, and soybean oil; and Halwa, a sugary food prepared with wheat flour, lentils, molasses, and soybean oil) provided 145–245 kcal/100 g^25,41,42^.

In the outpatient phase, Malawian children received RUTF rations biweekly through the Outpatient Feeding Program of Malawi^41^. In Bangladesh, caregivers were advised to prepare Khichuri and Halwa at home, and no food subsidies were provided other than micronutrient supplementation. Caregivers attended health education sessions during the rehabilitation phases, where they received hands-on training regarding the preparation of Khichuri and Halwa at home as part of Dhaka hospital guidelines. Caregivers were advised to have follow-up visits at D14 and D45, then monthly until the children reached a WLZ ≥ − 1^43^. Additionally, enrolled children visited the study facilities at D90 and D180 after discharge, following the CHAIN protocol.

### Sample size calculations

The sample size was based on work by Rising and Sonmez which evaluated energy expenditure in outpatient infants with malnutrition (EE, 101.3 ± 20 kcal/kg/day) and in community children (EE, 78.7 ± 8.4 kcal/kg/day)^14^. We estimated a conservative mean difference in mREE of 13.6 kcal/kg/day between children hospitalized with or without SM (which is 60% of the effect size reported by Rising and Sonmez) with a standard deviation of ± 14.2 kcal/kg/day. To detect this mean difference with a 5% Type-1 error rate and 80% power, we calculated that 22 children per group would be sufficient accounting for 10% attrition and 5% loss due to procedural challenges. Thus, we aimed to recruit at least 88 children split between the four groups.

### Indirect calorimetry (IC)

**Figure 5:**
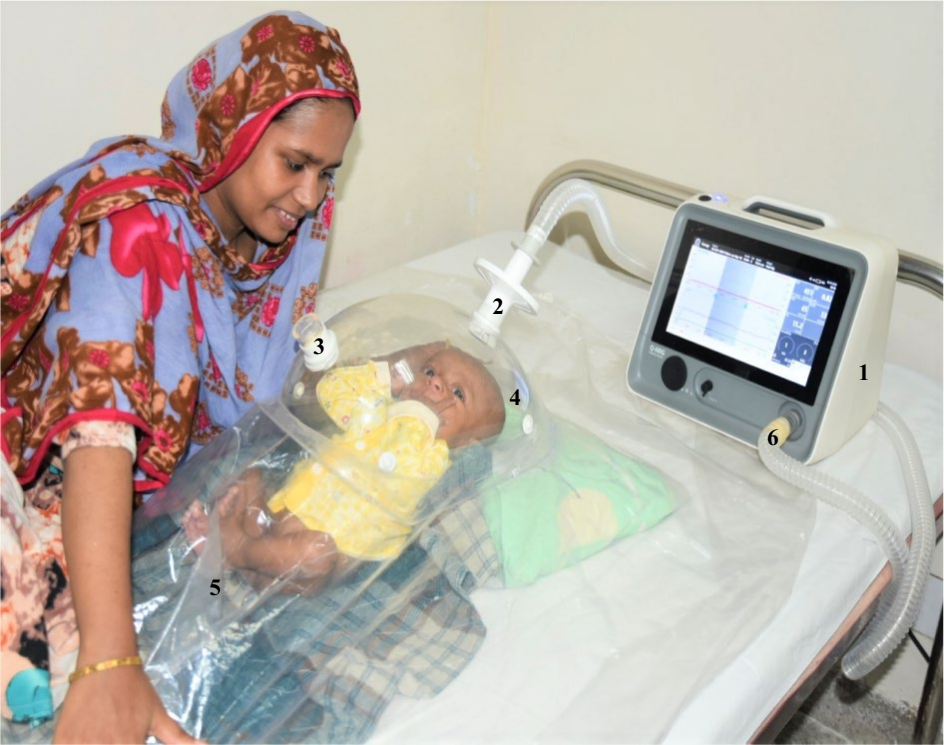
Measurement of gas exchange and resting energy expenditure using indirect calorimeter. Using the QNRG portable metabolic monitor (COSMED, Rome, Italy) (1); Patients exhale gases inside the ‘Canopy hood’ (4) diluted with a known airflow of ambient gas (3) of known concentration (determined prior to each session). The air circulates to the sensors through the canopy inlet with unidirectional air flow (6), and changes in O2 and CO2 caused by patient breathing are detected between in-flow and out-flow gases (i.e. VO2 and VCO2). From this, is derived the respiratory quotient (RQ, VO2/VCO2) and REE (kcal/day) as calculated by the Weir equation. Each measurement session utilizes a single-use veil (5) and an anti-bacterial filter (2). The photograph was taken and presented after obtaining written informed consent from the mother.

REE was measured (mREE) by IC at four time-points: (i) within 72 h of hospital admission (A); (ii) within 72 h from discharge (D0) (i.e. discharged participants were transferred to a research ward and observed for an additional four days); and after (iii) 14-days (D14) and (iv) 45-days (D45) post-discharge (within a ± 3-day follow-up window). IC was performed using the Q-NRG® respiratory indirect calorimeter (COSMED, Rome, Italy), which utilizes an open-circuit system for spontaneously breathing patients (Fig. 5).

The accuracy and precision of this device have been validated against mass spectrometry^44,45^ and the feasibility of bedside use in intensive care units has been confirmed^46^. Before each IC measurement, flow sensor calibration was performed, and child anthropometry and temperature were recorded. IC was not performed if the axillary temperature was ≥ 38 °C. Procedures were explained and demonstrated to the primary caregiver, who participated in preventing sleep and anxiety and minimizing child movement using colorful toys or screens. According to the operative's manual, children were placed under a transparent canopy hood with a disposable veil. The device had a dilution blower with a unidirectional airflow system into the canopy hood. We adjusted the blower to achieve a fractional expired CO_2_ gas concentration between 0.5 and 1.2%. Recordings are made every 30 s, and the first five measurements are discarded until a steady state of oxygen consumption (VO2) and carbon dioxide production (VCO2) is achieved with a coefficient of variation for both VO2 and VCO2 of ≤ 10% over five consecutive measurements^47^. The RQ was derived from the ratio of produced VCO2 divided by consumed VO2. RQ is a validity indicator for IC, which also reflects the dominant substrate used by the body. The average RQ representing mostly fat metabolism is 0.7, [RQ < 0.7 might suggest underfeeding and use of ketones], for mostly protein RQ, is ~ 0.8, and values near 1 suggest almost exclusive reliance on carbohydrates. Implausible IC measures were removed based on the following quality control cut-offs: (1) RQ of < 0.67 or > 1.1 and (2) having > 10% variability in either VCO2 or VO2. mREE (kcal/day) was then calculated using the Weir equation^48,49^. For weight correction, mREE was divided by child weight at each time point and expressed as kcal/kg/day. For children with edema, we used their lowest weight recorded during hospital admission to estimate weight corrected REE.

If the child remained calm, the procedure took approximately 12–15 min. In case of unsuccessful IC, the process was repeated after 2–3 h and, if necessary, the next day. Gas sensor calibration was performed monthly using a reference gas mixture (16% O_2_, 5% CO_2_, and balanced N_2_), and all study staff underwent extensive training.

### Predictive equations

Three different equations were used to estimate predicted REE (pREE): the WHO equation^13^, the weight-based Schofield (SchoWt) equation, and the weight and height-based Schofield (SchoWtHt) equation (Supplementary Table S1). These equations use simple predictors (age group, sex, weight, and height)^1^, are commonly applied and are recommended by pediatric scientific societies^23^.

### Statistical analyses

Determinants to be associated with mREE were pre-selected based on biological plausibility and literature review, and include: demographic variables (i.e. age, sex, site) and clinical variables such as nutritional group at admission, sepsis (i.e. confirmed or suspected infection with features of systemic inflammatory response syndrome [SIRS] in the absence of dehydration)^50^, SIRS score (i.e. abnormal body temperature [rectal temperature > 38.5 °C or < 35.0 °C], tachycardia, and abnormal white blood cell counts [WBC] or altered mental status); fever (axillary temperature ≥ 38 °C), diarrhea (i.e. having ≥ 3 loose or watery stools within past 24 h), dehydration, pneumonia (i.e. presence of cough or breathing difficulty considering age-specific fast breathing and chest in-drawing), anemia (i.e. haemoglobin < 11g/dl), WBC, malaria, HIV status [classified as: infected (or exposed if age ≤ 18 months) or non-infected (or non-exposed if age ≤ 18 months], duration of hospital stay. Spearman correlation tests were used to relate body weight and mREE across all timepoints. Two-level hierarchical models were fit to repeated measures of mREE clustered by child. Models included random intercepts per participant and unstructured covariance error. Random slopes were omitted based on singularity and fit parameters. Time was treated as weeks since admission but binned within time points (i.e. admission (A), 0 weeks; discharge (D0), 0.71 weeks (i.e. average length of hospital stay); 14-days post-discharge (D14), 2.71 weeks; 45-days post-discharge (D45), 7.14 weeks. Linear and non-linear fits were evaluated based on Akaike’s information criterion (AIC) and Bayesian information criteria (BIC) (including polynomial, quadratic and piecewise models fit with a single knot at discharge). The base model included time, nutritional group, their interaction and age as predictors (M_0_: REE ~ time × nutritional group + age). Marginal means were calculated for each group and time point and specified contrasts tested. Slopes before and after discharge were derived and tested for differences using linear combinations of the piecewise model coefficients. Additional models were built to assess association with clinical features, where nested models were fit with maximum likelihood, compared, and selected for parsimony based on AIC and BIC. For sensitivity testing, other models were further adjusted for potential confounders (i.e. sex, site, duration of hospital stay). Multicollinearity was assessed (i.e. variance inflation factor < 5) and final models were fit using restricted maximum likelihood.

To determine the accuracy of the three predictive equations, we calculated for each patient the difference in kcal/day between mREE and pREE. The average error bias per equation, group and time point is presented as the percent difference [(pREE − mREE)/mREE × 100]. The number and percentage of subjects with over- or under- estimated pREE was calculated considering the accepted accuracy cut-off of ± 10% of mREE. For each equation, Bland–Altman plots were also generated and discrepancy between pREE and mREE were tested for differences over time and between groups using mixed models. To compare measured energy requirements to current clinical feeding targets used for severely malnourished children, we calculated for each patient the lower and upper range of calories that would be prescribed based on patient weight. We then described the differences between mREE and either the low- or upper- clinical feeding targets using mixed models. To compare measured energy requirements to current clinical feeding targets used for severely malnourished children, we calculated for each patient the lower and upper range of calories that would be prescribed based on patient weight. We then described the differences between mREE and either the low- or upper- clinical feeding targets using mixed models. For calculations regarding stabilization phase, weights and mREE at admission were used, whereas estimates for the rehabilitation phase were based on discharge measures, and 45-days post-discharge values were used to represent the post-discharge period.

Our measured REE may have been slightly higher because a complete fasted state was not achieved in malnourished children due to the risk of hypoglycemia. It was not feasible to make meaningful corrections to REE for energy expended due to tissue synthesis and the thermic effect of food. Consequently, we performed a sensitivity analysis to determine whether Dietary-Induced Thermogenesis (DIT) is responsible for the observed difference between mREE and pREE. To eliminate the effect of DIT, we reduced the mREE by 10%, considering that DIT represents only 5–10% of TEE.

Statistical analysis was performed using IBM SPSS Statistics 25, STATA version 13, and R (version 4.0.2). For analysis and reporting, we followed the Strengthening the Reporting of Observational Studies in Epidemiology (STROBE) reporting guideline (Supplementary information).

### Ethics declarations

Institutional review boards including the University of Oxford Tropical Ethics Committee, icddr,b, the QECH and the Hospital for Sick Children, Toronto, Canada approved the study. Written informed consent was obtained from parents or caregivers of all participants before enrollment.

### Supplementary Information

Supplementary Information.

## Data Availability

The dataset is deposited at the Harvard Dataverse and can be requested from the website.
