# Supplementary material for: Trajectories of resting energy expenditure and performance of predictive equations in children hospitalized with an acute illness and malnutrition: a longitudinal study
Source: Sci Rep. 2024 Feb 13;14:3613. doi: 10.1038/s41598-024-53791-w (PMC10864294; doi:10.1038/s41598-024-53791-w)
Supplement: Supplementary file 1 — Supplementary Information. [file 41598_2024_53791_MOESM1_ESM.pdf]

Supplementary Information

Trajectories of Resting Energy Expenditure and Performance of Predictive Equations in Children Hospitalized with an Acute Illness and Malnutrition: a longitudinal study

Farzana Afroze, Farnaz Khoshnevisan, Philliness Prisca Harawa, Zahidul Islam, Celine Bourdon, Stanley Khoswe, Munirul Islam, Shafiqul Alam Sarker, Farhana Islam, Abu Sadat Mohammad Sayeem Bin Shahid, Koen Joosten, Jessie M Hulst, Chisomo Eneya, Judd L Walson, James A Berkley, Isabel Potani, Wieger Voskuijl, Tahmeed Ahmed, Mohammod Jobayer Chisti, Robert H J Bandsma

Contents

Supplementary Table S1. Algorithms of the three equations used to calculate predicted REE in this study.

Supplementary Table S2. Anthropometry and weight-corrected REE (kcal/kg/day) and RQ values measured in ill children with different nutritional status at each time point.

Supplementary Table S3. Parameter estimates of resting energy expenditure (kcal/kg/day) trajectories overtime in children hospitalized for an acute illness with differing nutritional status derived from piecewise mixed effect models.

Supplementary Table S4. Parameter estimates testing association of resting energy expenditure (kcal/kg/day) trajectories overtime in children hospitalized for an acute illness with differing nutritional status with clinical variables of stunting and dehydration.

Supplementary Table S5. Parameter estimates testing association of resting energy expenditure (kcal/kg/day) trajectories overtime in children hospitalized for an acute illness with differing nutritional status with clinical variables of sepsis, high SIRS score and anemia.

Supplementary Table S6. Parameter estimates testing association of resting energy expenditure (kcal/kg/day) trajectories overtime in children hospitalized for an acute illness with differing nutritional status with clinical variables of pneumonia, diarrhea, fever and WBC (109L).

Supplementary Table S7. Bias between measured and predicted resting energy expenditure as estimated by 3 equations in children hospitalized with acute illness and different nutritional status at each time point.

Supplementary Table S8. Differences between the measured resting energy expenditure accounting for non-fasting state and predicted resting energy expenditure as estimated by three equations in children hospitalized with acute illness at each time point.

Supplementary Figure S1. Study flow chart of recruited participants.

Supplementary Figure S2. Venn diagram detailing number and percent of children diagnosed with more than one clinical condition.

Supplementary Figure S3. Relationship between body weight (kg) and REE (kcal/day) of children represented split by nutritional status as measured with indirect calorimetry.

Supplementary Figure S4. Resting energy expenditure (REE, kcal/kg/day) of children with edematous malnutrition at admission (A) and discharge (D0).

Supplementary Figure S5. Regression coefficients derived from piecewise mixed models testing the association between resting energy expenditure and different clinical diagnoses in children hospitalized with acute illness and of varying nutritional status.

Supplementary Figure S6. Bland-Altman plots comparing absolute differences between measured REE using indirect calorimetry and predicted REE (Y axis), and average of measured and predicted REE (X axis) for the three predictive equations

STROBE Statement—Checklist of items that should be included in reports of cohort studies.

Supplementary Table S1. Algorithms of the three equations used to calculate predicted REE in this study.

	**Abbreviation**	**Gender**	**Age**	**Equation**
WHO	WHO pREE	M	<3 years	(60.9 × weight) - 54
		F	<3 years	(61.0 × weight) - 51
Schofield with weight correction	SchoWt pREE	M	<3 years	(50.48 × weight) - 30.33
		F	<3 years	(58.29 × weight) - 31.05
Schofield with weight correction	SchoWtHt pREE	M	<3 years	(0.167 × weight) + (1517.4 × height) - 617.6
		F	<3 years	(16.25 × weight) + (1023.2 × height) - 413.5

All equations are in kcal\day, weight in kg, height in meters. pREE, predicted resting energy expenditure.

Supplementary Table S2. Anthropometry and weight-corrected REE (kcal/kg/day) and RQ values measured in ill children with different nutritional status at each time point.

		**NW**<br>n=23	**MW**<br>n=29	**SW**<br>n = 51	**EM**<br>n = 22
Admission	Weight	8.2 (7.6, 9.1)	6.6 (6.0, 7.0)	5.1 (4.4, 6.2)	6.9 (6.1, 8.2)
	Height	71 (69, 74)	67 (64, 70)	64 (58, 70)	72 (63, 75)
	MUAC	13.6 (13.1, 14.4)	12.1 (12, 12.3)	10.7 (10.2, 11.3)	12.4 (11.2, 13.0)
	WLZ	-0.50 (-1.1, 0.10)	-2.0 (-2.4, -1.6)	-3.4 (-3.8, -2.6)	-1.4 (-2.7, -0.57)
	WAZ	-1.5 (-1.9, -0.22)	-2.7 (-3.2, -1.9)	-4.2 (-4.7, -3.5)	-2.8 (-3.6, -1.7)
	LAZ	-1.8 (-2.2, -0.95)	-1.7 (-3.0, -0.73)	-3.1 (-4.2, -2.1)	-2.9 (-3.4, -2.5)
	REE	64.0 (57.2, 74.0) (n=14)	68.6 (66.6, 80.1) (n=26)	79.7 (67.8, 89.6) (n=40)	62.4 (56.5, 69.6) (n=15)
	RQ	0.74 (0.69, 0.80)	0.76 (0.71, 0.81)	0.74 (0.72, 0.80)	0.77 (0.71, 0.86)
Discharge	Weight	8.2 (7.5, 9.3)	6.6 (6.0, 7.0)	5.5 (4.5, 6.4)	7.2 (5.6, 8.2)
	Height	71 (68, 74)	68 (65, 70)	65 (58, 70)	72 (63, 75)
	MUAC	13.5 (13.1, 14.3)	12.2 (12.0, 12.3)	10.7 (10.1, 11.7)	12.2 (11.4, 12.9)
	WLZ	-0.34 (-0.90, 0.01)	-2.0 (-2.4, -1.6)	-2.8 (-3.6, -1.8)	-1.7 (-2.9, -0.97)
	WAZ	-1.4 (-1.9, -0.14)	-2.5 (-3.1, -2.0)	-3.8 (-4.7, -3.3)	-2.9 (-3.8, -2.2)
	LAZ	-1.7 (-2.3, -0.79)	-1.8 (-3.4, -0.80)	-3.3 (-4.1, -2.1)	-2.9 (-3.4, -2.5)
	REE	63.9 (58.2, 69.4) (n=21)	71.5 (60.2, 85.6) (n=25)	82.0 (74.6, 92.8) (n=45)	77.4 (63.2, 92.5) (n=21)
	RQ	0.80 (0.76, 0.86)	0.78 (0.74, 0.87)	0.80 (0.74, 0.84)	0.85 (0.79, 0.88)
14-days	Weight	8.1 (7.8, 9.1)	6.6 (6.2, 7.1)	5.5 (4.7, 6.7)	7.7 (6.2, 90.)
	Height	72 (70, 74)	68 (65, 70)	65 (59, 70)	72 (65, 76)
	MUAC	13.6 (13.5, 14.8)	12.2 (11.9, 12.6)	11.3 (10.7, 11.7)	12.6 (11.5, 13.9)
	WLZ	-0.67 (-1.3, 0.32)	-2.0 (-2.4, -1.4)	-2.5 (-3.5, -1.3)	-1.1 (-2.1, 0.02)
	WAZ	-1.4 (-1.9, -0.67)	-2.7 (-3.4, -2.1)	-3.7 (-4.5, -3.1)	-2.3 (-3.4, -1.4)
	LAZ	-2.0 (-2.3, -0.78)	-2.2 (-3.6, -1.1)	-3.3 (-4.4, -2.2)	-3.0 (-3.7, -2.4)
	REE	67.3 (57.0, 76.8) (n=16)	81.6 (66.1, 92.5) (n=21)	77.2 (67.8, 94.4) (n=41)	71.1 (64.7, 89.0) (n=18)
	RQ	0.80 (0.76, 0.81)	0.79 (0.76, 0.82)	0.80 (0.75, 0.85)	0.82 (0.78, 0.88)
45-days	Weight	8.9 (8.2, 9.8)	7.3 (6.9, 7.6)	6.1 (5.2, 6.8)	8.1 (7.3, 9.9)
	Height	74 (71, 76)	69 (67, 71)	66 (60, 70)	73 (65, 76)
	MUAC	14.0 (13.5, 14.8)	12.9 (12.1, 13.3)	11.6 (11.0, 12.2)	13.4 (12.7, 14.7)
	WLZ	-0.28 (-0.83, 0.50)	-1.4 (-1.7, -0.97)	-2.3 (-3.0, -1.0)	-0.29 (-1.1, 0.58)
	WAZ	-0.76 (-1.6, 0.09)	-2.0 (-3.1, -1.5)	-3.6 (-4.1, -2.8)	-1.7 (-3.0, -0.71)
	LAZ	-1.5 (-2.4, -0.53)	-2.3 (-3.4, -0.86)	-3.4 (-4.4, -2.3)	-2.9 (-3.5, -2.2)
	REE	64.2 (57.1, 71.8) (n=19)	70.7 (65.0, 86.4) (n=21)	83.6 (74.0, 93.2) (n=41)	80.3 (58.0, 93.7) (n=17)
	RQ	0.78 (0.76, 0.82)	0.76 (0.73, 0.78)	0.79 (0.74, 0.82)	0.81 (0.79, 0.89)

Median (IQR) values for anthropometry, resting energy expenditure (REE) and respiratory quotient (RQ). MUAC- mid-upper arm circumference, WLZ- weight-for-length z-score, WAZ- weight-for-age z-score, LAZ- length-for-age z-score, NW- no wasting, MW- moderate wasting, SW- severe wasting; EM- edematous malnutrition.

Supplementary Table S3. Parameter estimates of resting energy expenditure (kcal/kg/day) trajectories overtime in children hospitalized for an acute illness with differing nutritional status derived from piecewise mixed effect models.

	Est.	***CI***	***p***
Intercept [NW]	73.2	63.4, 83.0	**<0.001**
Slope during admission [NW]	2.94	-9.63, 15.5	0.65
Change of slope during admission to post-discharge [NW]	-3.47	-16.6, 9.67	0.60
Difference in intercept [MW - NW]	2.88	-7.90, 13.7	0.60
Difference in intercept [SW - NW]	12.2	2.15, 22.3	**0.017**
Difference in intercept [EM - NW]	-1.92	-13.9, 10.0	0.75
Difference in slope during admission [MW - NW]	3.91	-12.1, 20.0	0.63
Difference in slope during admission [SW - NW]	1.06	-13.7, 15.8	0.89
Difference in slope during admission [EM - NW]	17.1	-0.42, 34.7	0.056
Difference in change of slope during admission to post-discharge [MW - NW]	-3.52	-20.4, 13.3	0.68
Difference in change of slope during admission to post-discharge [SW - NW]	-0.25	-15.7, 15.2	0.98
Difference in change of slope during admission to post-discharge [EM - NW]	-16.7	-35.1, 1.68	0.075
Age, months	-0.66	-1.08, -0.24	**0.0020**
**Random Effects**			
σ^2	185		
$\tau_{00\ record\_id}$	103		
ICC	0.36		
N $_{record\_id}$	125		
Observations	401		
Marginal R^2 / Conditional R^2	0.16 / 0.46		

Piecewise mixed models were fitted with a single knot positioned at discharge, and with random intercepts per participant allowing to evaluate the intercepts, slopes, and differences in slopes between groups during hospitalization (i.e., during admission) versus post-discharge. Time was coded as weeks since admission binned within time points. Final models were fit using restricted maximum likelihood. Children with NW- no wasting (reference group), MW-moderate wasting, SW- severe wasting, or EM- edematous malnutrition.

Supplementary Table S4. Parameter estimates testing association of resting energy expenditure (kcal/kg/day) trajectories overtime in children hospitalized for an acute illness with differing nutritional status with clinical variables of stunting and dehydration.

	M_0: Base			M_1: Base + Stunting			M_2: Base + Dehydration		
	Est.	***95%CI***	***p***	***Est.***	***95%CI***	***p***	***Est.***	***95%CI***	***p***
Intercept [NW]	73	63, 83	**<0.001**	72	62, 81	**<0.001**	70	61, 81	**<0.001**
Slope during admission [NW]	2.9	-9.6, 16	0.64	3.0	-9.6, 16	0.64	3.1	-9.4, 16	0.63
Change of slope during admission vs post-discharge [NW]	-3.5	-17, 9.7	0.60	-3.5	-17, 9.6	0.60	-3.6	-17, 9.5	0.59
Difference in intercept [MW - NW]	2.9	-7.9, 14	0.60	1.4	-9.2, 12	0.80	1.4	-9.4, 12	0.80
Difference in intercept [SW - NW]	12	2.2, 22	**0.017**	7.5	-2.7, 18	0.15	11	0.92, 21	**0.032**
Difference in intercept [EM - NW]	-1.9	-14, 10	0.75	-5.9	-18, 5.9	0.33	-2.8	-15, 9.0	0.63
Difference in slope during admission [MW - NW]	3.9	-12, 20	0.63	3.6	-12, 20	0.66	4.2	-12, 20	0.60
Difference in slope during admission [SW - NW]	1.1	-14, 16	0.89	0.95	-14, 16	0.90	1.2	-14, 16	0.87
Difference in slope during admission [EM - NW]	17	-0.42, 35	0.056	17	-0.63, 34	0.059	17	-0.34, 35	0.055
Difference in change of slope during admission vs post-discharge [MW - NW]	-3.5	-20, 13	0.68	-3.3	-20, 14	0.71	-3.8	-21, 13	0.66
Difference in change of slope during admission vs post-discharge [SW - NW]	-0.25	-16, 15	0.98	-0.17	-16, 15	0.98	-0.39	-16, 15	0.96
Difference in change of slope during admission vs post-discharge [EM - NW]	-17	-35, 1.7	0.075	-17	-35, 1.9	0.078	-17	-35, 1.6	0.073
Age, months	-0.66	-1.1, -0.24	**0.002**	-0.72	-1.1, -0.31	**0.001**	-0.54	-0.97, -0.12	**0.012**
Clinical variable [Yes] (for stunting [Moderate])				6.9	1.2, 13	**0.018**	6.3	1.5, 11	**0.011**
Clinical variable [Severe]				11	5.0, 16	**<0.001**			
**Random Effects**									
σ^2	184			185			184		
τ_{00}	103 $_{\text{record\_id}}$			86 $_{\text{record\_id}}$			97 $_{\text{record\_id}}$		
ICC	0.36			0.32			0.35		
N	125 $_{\text{record\_id}}$			125 $_{\text{record\_id}}$			125 $_{\text{record\_id}}$		
Observations	401			401			401		
Marginal R^2 / Conditional R^2	0.16 / 0.46			0.21 / 0.46			0.18 / 0.47		

Piecewise mixed models were fitted with a single knot positioned at discharge and random intercepts per participant allowing to evaluate the intercepts, slopes, and differences in slopes between groups during hospitalization (i.e., during admission) versus post-discharge. Time was coded as weeks since admission binned within time points. Final models were fit using restricted maximum likelihood. Children with NW- no wasting (reference group), MW-moderate wasting, SW- severe wasting, or EM- edematous malnutrition.

Supplementary Table S5. Parameter estimates testing association of resting energy expenditure (kcal/kg/day) trajectories overtime in children hospitalized for an acute illness with differing nutritional status with clinical variables of sepsis, high SIRS score and anemia.

	**M_3: Base + Sepsis**			**M_4: Base + High SIRS score**			**M_5: Base + Anemia**		
	Est.	***95%CI***	***p***	***Est.***	***95%CI***	***p***	***Est.***	***95%CI***	***p***
Intercept [NW]	76	66, 85	**<0.001**	78	68, 89	**<0.001**	73.6	63, 84	**<0.001**
Slope during admission [NW]	2.5	-10, 15	0.69	2.4	-10, 15	0.71	2.9	-9.6, 16	0.65
Change of slope during admission vs post-discharge [NW]	-3.1	-16, 10	0.65	-2.9	-16, 10	0.66	-3.5	-17, 9.7	0.60
Difference in intercept [MW - NW]	1.0	-9.7, 12	0.85	0.44	-10, 11	0.94	3.1	-7.8, 14	0.58
Difference in intercept [SW - NW]	11	1.4, 21	**0.026**	10	0.19, 20	**0.046**	12.	2.2, 22	**0.017**
Difference in intercept [EM - NW]	-2.3	-14, 9.5	0.70	-3.4	-15, 8.5	0.58	-1.9	-14, 10	0.75
Difference in slope during admission [MW - NW]	4.6	-11, 21	0.57	4.1	-12, 20	0.61	3.9	-12, 20	0.63
Difference in slope during admission [SW - NW]	1.8	-13, 17	0.81	1.4	-13, 16	0.85	1.1	-14, 16	0.89
Difference in slope during admission [EM - NW]	18	0.29, 35	**0.046**	17	-0.14, 35	0.052	17	-0.44, 35	0.056
Difference in change of slope during admission vs post-discharge [MW - NW]	-4.2	-21, 13	0.62	-3.7	-21, 13	0.66	-3.5	-20, 13	0.68
Difference in change of slope during admission vs post-discharge [SW - NW]	-0.96	-16, 15	0.90	-0.63	-16, 15	0.94	-0.26	-16, 15	0.97
Difference in change of slope during admission vs post-discharge [EM - NW]	-17	-36, 1.0	0.064	-17	-35, 1.4	0.071	-17	-35, 1.7	0.075
Age, months	-0.70	-1.1, -0.29	**0.001**	-0.80	-1.2, -0.37	**<0.001**	-0.67	-1.1, -0.24	**0.002**
Clinical variable [Yes]	-9.6	-16, -2.9	**0.005**	-6.6	-12, -1.4	**0.014**	-0.66	-5.5, 4.2	0.79
**Random Effects**									
σ^2	184			185			186		
τ_{00}	93 $_{record\_id}$			95 $_{record\_id}$			104 $_{record\_id}$		
ICC	0.34			0.34			0.36		
N	125 $_{record\_id}$			125 $_{record\_id}$			125 $_{record\_id}$		
Observations	401			401			401		
Marginal R^2 / Conditional R^2	0.19 / 0.46			0.18 / 0.46			0.16 / 0.46		

Piecewise mixed models were fitted with a single knot positioned at discharge and random intercepts per participant allowing to evaluate the intercepts, slopes, and differences in slopes between groups during hospitalization (i.e., during admission) versus post-discharge. Time was coded as weeks since admission binned within time points. Final models were fit using restricted maximum likelihood. Children with NW- no wasting (reference group), MW-moderate wasting, SW- severe wasting, or EM- edematous malnutrition.

Supplementary Table S6. Parameter estimates testing association of resting energy expenditure (kcal/kg/day) trajectories overtime in children hospitalized for an acute illness with differing nutritional status with clinical variables of pneumonia, diarrhea, fever and WBC (10^9L).

	**M_6: Base + Pneumonia**			**M_7: Base + Diarrhea**			**M_8: Base + Fever**			**M9: Base + WBC**		
	Est.	***95% CI***	***p***	***Est.***	***95% CI***	***p***	***Est.***	***95% CI***	***p***	***Est.***	***95% CI***	***p***
Intercept [NW]	72	62, 82	**<0.001**	72	61, 82	**<0.001**	74	63, 83	**<0.001**	85	71, 99	**<0.001**
Slope during admission [NW]	2.8	-9.7, 15	0.66	3.0	-9.5, 16	0.63	3.0	-9.6, 16	0.65	3.3	-10, 17	0.63
Change of slope during admission vs post-discharge [NW]	-3.4	-17, 9.8	0.61	-3.6	-17, 9.6	0.60	-3.5	-17, 9.7	0.61	-3.2	-17, 11	0.65
Difference in intercept [MW - NW]	3.1	-7.7, 14	0.57	1.8	-9.3, 13	0.76	2.6	-8.3, 14	0.64	0.32	-11, 11	0.96
Difference in intercept [SW - NW]	12	2.1, 22	**0.018**	11	1.1, 21	**0.030**	12	1.7, 22	**0.022**	14	2.9, 24	**0.013**
Difference in intercept [EM - NW]	-2.4	-14, 9.6	0.69	-2.5	-15, 9.5	0.68	-2.2	-14, 9.9	0.72	-2.3	-15, 11	0.73
Difference in slope during admission [MW - NW]	4.0	-12, 20	0.62	4.0	-12, 20	0.63	4.0	-12, 20	0.63	3.5	-13, 20	0.68
Difference in slope during admission [SW - NW]	1.1	-14, 16	0.89	1.0	-14, 16	0.89	1.1	-14, 16	0.88	-1.2	-17, 15	0.88
Difference in slope during admission [EM - NW]	17	-0.14, 35	0.052	17	-0.72, 34	0.060	17	-0.40, 35	0.056	15	-4.4, 34	0.13
Difference in change of slope during admission vs post-discharge [MW - NW]	-3.6	-21, 13	0.67	-3.6	-20, 13	0.68	-3.6	-20, 13	0.68	-3.7	-21, 14	0.68
Difference in change of slope during admission vs post-discharge [SW - NW]	-0.26	-16, 15	0.97	-0.24	-16, 15	0.98	-0.29	-16, 15	0.97	1.4	-15, 18	0.87
Difference in change of slope during admission vs post-discharge [EM - NW]	-17	-35, 1.40	0.070	-16	-35, 2.0	0.080	-17	-35, 1.7	0.075	-15	-36, 4.9	0.14
Age, months	-0.62	-1.1, -0.19	**0.005**	-0.61	-1.0, -0.18	**0.006**	-0.65	-1.1, -0.23	**0.003**	-0.88	-1.4, -0.41	**<0.001**
Clinical variable [Yes]	2.6	-2.5, 7.6	0.32	2.4	-3.4, 8.2	0.42	-0.87	-6.9, 5.1	0.78	-0.52	-0.96, -0.08	**0.022**
**Random Effects**												
σ^2	185			185			185			181		
τ_{00}	102 $_{record\_id}$			103 $_{record\_id}$			104 $_{record\_id}$			95 $_{record\_id}$		
ICC	0.36			0.36			0.36			0.34		
N	125 $_{record\_id}$			125 $_{record\_id}$			125 $_{record\_id}$			102 $_{record\_id}$		
Observations	401			401			401			337		
Marginal R^2 / Conditional R^2	0.16 / 0.46			0.16 / 0.46			0.16 / 0.46			0.19 / 0.47		

Piecewise mixed models were fitted with a single knot positioned at discharge and random intercepts per participant allowing to evaluate the intercepts, slopes, and differences in slopes between groups during hospitalization (i.e., during admission) versus post-discharge. Time was coded as weeks since admission binned within time points. Final models were fit using restricted maximum likelihood. Children with NW- no wasting (reference group), MW-moderate wasting, SW- severe wasting, or EM- edematous malnutrition

Supplementary Table S7. Bias between measured and predicted resting energy expenditure as estimated by 3 equations in children hospitalized with acute illness and different nutritional status at each time point.

	**NW n=23**				**MW n=29**				**EM n=22**				**SW n=51**			
	**REE**	**Bias**	**Over**	**Under**	**REE**	**Bias**	**Over**	**Under**	**REE**	**Bias**	**Over**	**Under**	**REE**	**Bias**	**Over**	**Under**
**Admission**																
Measured REE (kcal/d)	532 (467, 632)	NA	NA	NA	452 (389, 490)	NA	NA	NA	400 (350, 525)	NA	NA	NA	397 (356, 459)	NA	NA	NA
WHO	446 (408, 498)	-17%	1 (7.1%)	13 (93%)	348 (312, 367)	-25%	4 (15%)	22 (85%)	369 (316, 451)	-14%	2 (13%)	13 (87%)	260 (216, 321)	-38%	1 (2.5%)	39 (98%)
Schofield weight	458 (421, 507)	-14%	1 (7.1%)	13 (93%)	358 (327, 375)	-23%	4 (15%)	22 (85%)	375 (331, 449)	-11%	3 (20%)	12 (80%)	273 (230, 334)	-35%	1 (2.5%)	39 (98%)
Schofield weight and height	466 (425, 502)	-18%	1 (7.1%)	13 (93%)	401 (342, 423)	-14%	5 (19%)	21 (81%)	445 (331, 500)	-11%	4 (27%)	11 (73%)	342 (263, 436)	-19%	7 (18%)	33 (82%)
**Discharge**																
Measured REE (kcal/d)	557 (439, 640)	NA	NA	NA	467 (424, 493)	NA	NA	NA	501 (419, 633)	NA	NA	NA	447 (342, 505)	NA	NA	NA
WHO	446 (406, 509)	-13%	3 (14%)	18 (86%)	349 (330, 374)	-26%	1 (4.0%)	24 (96%)	387 (284, 448)	-31%	1 (4.8%)	20 (95%)	281 (223, 342)	-37%	1 (2.2%)	44 (98%)
Schofield weight	457 (414, 520)	-10%	3 (14%)	18 (86%)	358 (342, 381)	-25%	1 (4.0%)	24 (96%)	394 (300, 455)	-30%	1 (4.8%)	20 (95%)	297 (236, 350)	-34%	1 (2.2%)	44 (98%)
Schofield weight and height	466 (423, 510)	-12%	4 (19%)	17 (81%)	405 (366, 430)	-18%	5 (20%)	20 (80%)	444 (332, 500)	-24%	2 (9.5%)	19 (90%)	348 (266, 435)	-24%	1 (2.2%)	44 (98%)
**14-days post-discharge**																
Measured REE (kcal/d)	551 (427, 602)	NA	NA	NA	489 (431, 591)	NA	NA	NA	548 (467, 596)	NA	NA	NA	460 (354, 523)	NA	NA	NA
WHO	439 (421, 502)	-19%	3 (19%)	13 (81%)	352 (324, 380)	-37%	2 (9.5%)	19 (90%)	415 (323, 497)	-24%	1 (5.6%)	17 (94%)	283 (234, 353)	-39%	2 (4.9%)	39 (95%)
Schofield weight	449 (429, 510)	-17%	3 (19%)	13 (81%)	363 (339, 391)	-34%	2 (9.5%)	19 (90%)	421 (338, 492)	-23%	1 (5.6%)	17 (94%)	299 (245, 365)	-36%	2 (4.9%)	39 (95%)
Schofield weight and height	468 (439, 514)	-17%	3 (19%)	13 (81%)	409 (369, 433)	-28%	3 (14%)	18 (86%)	448 (357, 510)	-26%	0 (0%)	18 (100%)	367 (281, 443)	-28%	4 (9.8%)	37 (90%)
**45-days post-discharge**																
Measured REE (kcal/d)	574 (512, 636)	NA	NA	NA	497 (434, 606)	NA	NA	NA	588 (490, 692)	NA	NA	NA	509 (434, 554)	NA	NA	NA
WHO	486 (450, 541)	-13%	0 (0%)	19 (100%)	395 (365, 407)	-25%	1 (4.8%)	20 (95%)	437 (390, 553)	-33%	0 (0%)	17 (100%)	316 (264, 364)	-39%	0 (0%)	41 (100%)
Schofield weight	497 (450, 551)	-13%	1 (5.3%)	18 (95%)	402 (375, 419)	-22%	1 (4.8%)	20 (95%)	449 (395, 546)	-33%	1 (5.9%)	16 (94%)	331 (274, 374)	-37%	0 (0%)	41 (100%)
Schofield weight and height	493 (449, 519)	-20%	1 (5.3%)	18 (95%)	426 (392, 447)	-20%	1 (4.8%)	20 (95%)	459 (377, 529)	-30%	2 (12%)	15 (88%)	380 (298, 453)	-30%	1 (2.4%)	40 (98%)

Median (IQR) of predicted REE estimated using 3 equations (i.e., WHO, Schofield weight, and Schofield weight and height). Percent bias is calculated as [(predicted REE-measured REE)/measured REE] *100. Number and percentage of children with REE over- or under- estimated by more than ±10%. REE- resting energy expenditure; NW, no wasting; MW, moderate wasting; SW, severe wasting; EM, edematous malnutrition. NA, not applicable.

Supplementary Table S8. Differences between the measured resting energy expenditure accounting for non-fasting state and predicted resting energy expenditure as estimated by three equations in children hospitalized with acute illness at each time point.

	**Resting energy expenditure**	**Percent bias**	**Over estimation**	**Under estimation**
**Admission**				
⸸Reduced measured REE kcal/day	409 (346, 465)	NA	NA	NA
WHO	335 (260, 412)	-16%	17 (13.6%)	78 (62.4%)
Schofield weight	347 (273, 415)	-14%	19 (15.2%)	75 (60%)
†Schofield weight and height	404 (320, 468)	-3%	32 (25.6%)	47 (37.6%)
**Discharge**				
Measured REE kcal/day	424 (370, 511)	NA	NA	NA
WHO	347 (266, 422)	-23%	11 (8.8%)	88 (70.4%)
Schofield weight	357 (272, 429)	-21%	12 (9.6%)	82 (65.6%)
Schofield weight and height	406 (327, 463)	-11%	18 (14.4%)	61 (48.8%)
**14-days post-discharge**				
Measured REE kcal/day	441 (387, 524)	NA	NA	NA
WHO	353 (279, 437)	-19%	10 (8%)	84 (67.2%)
Schofield weight	366 (290, 438)	-16%	12 (9.6%)	79 (63.2%)
Schofield weight and height	408 (332, 468)	-11%	18 (14.4%)	60 (48%)
**45-days post-discharge**				
Measured REE kcal/day	483 (413, 558)	NA	NA	NA
WHO	389 (316, 464)	-22%	11 (8.8%)	79 (63.2%)
Schofield weight	395 (329, 475)	-21%	11 (8.8%)	76 (60.8%)
Schofield weight and height	429 (346, 489)	-14%	14 (11.2%)	68 (54.4%)

Median (IQR) of predicted REE estimated using 3 equations (i.e., WHO, Schofield weight, and Schofield weight and height). Percent bias is calculated as [(predicted REE-measured REE)/measured REE] *100. ⸸The reduced measured resting energy expenditure (REE) was calculated by applying a 10% reduction to the REE measured by indirect calorimetry. †p-value is <0.05 for all equations except Schofield weight and height equation during admission (p= 0.104). NA, not applicable.

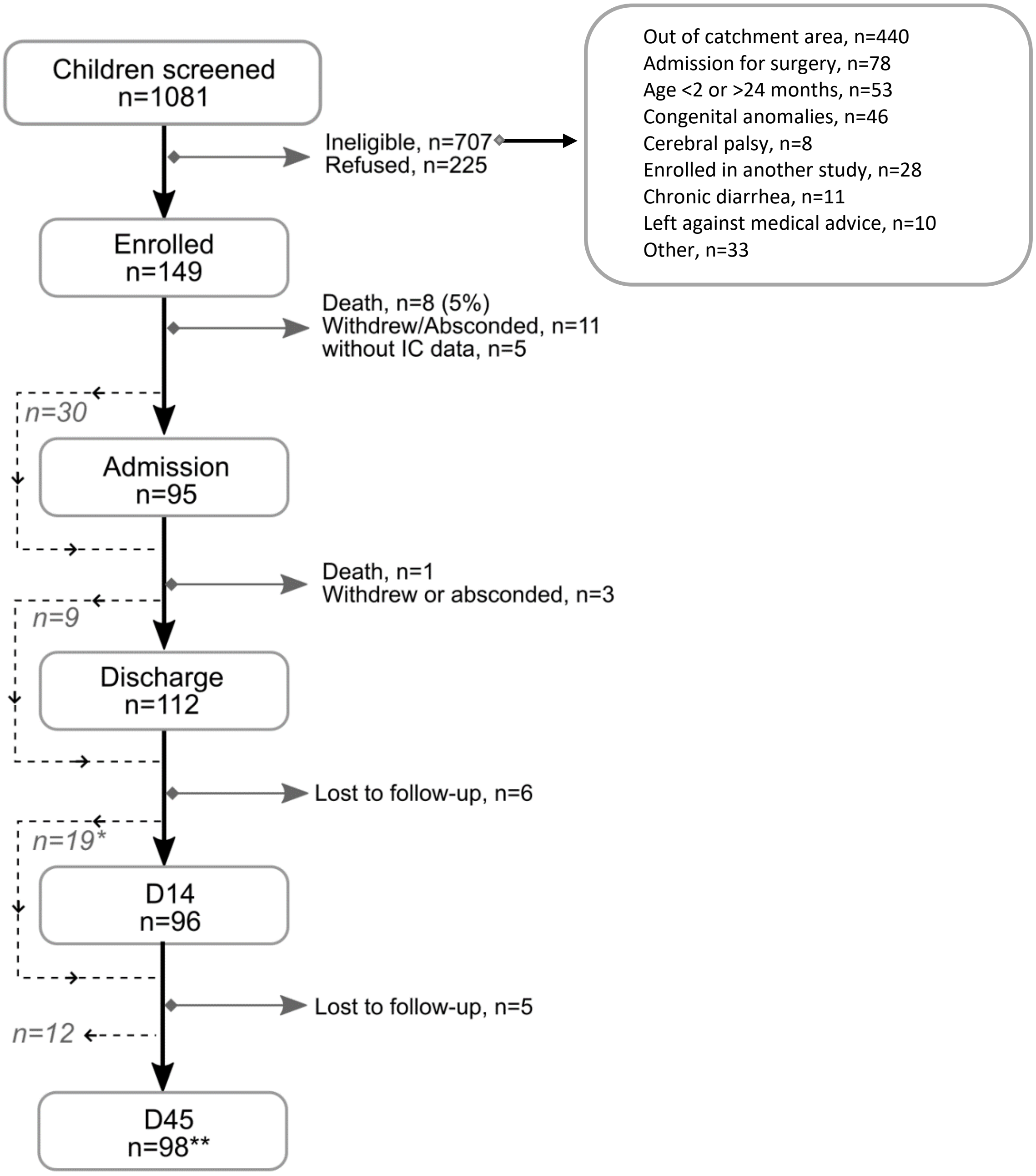

Supplementary Figure S1. Study flow chart of recruited participants. 125 children provided at least one valid IC-measure of REE. For each time point, dashed lines indicate number of participants for which IC was not successful. *Includes one child that completely missed the visit. **Includes one participant who died during the study but after 45-days post-discharge. IC, indirect calorimetry. D14, 14-days post discharge; D45, 45-days post discharge.

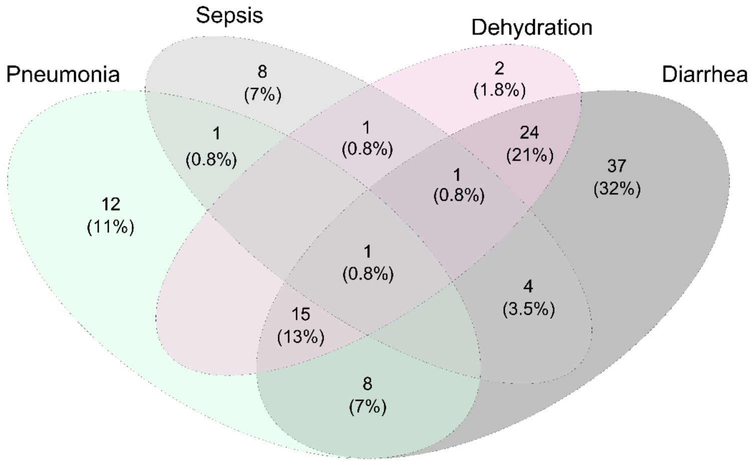

Supplementary Figure S2. Venn diagram detailing number and percent of children diagnosed with more than one clinical condition. The most commonly co-occurring diagnosis is dehydration and diarrhea (n=24, 21%); while 15 (13%) have dehydration, diarrhea, and pneumonia

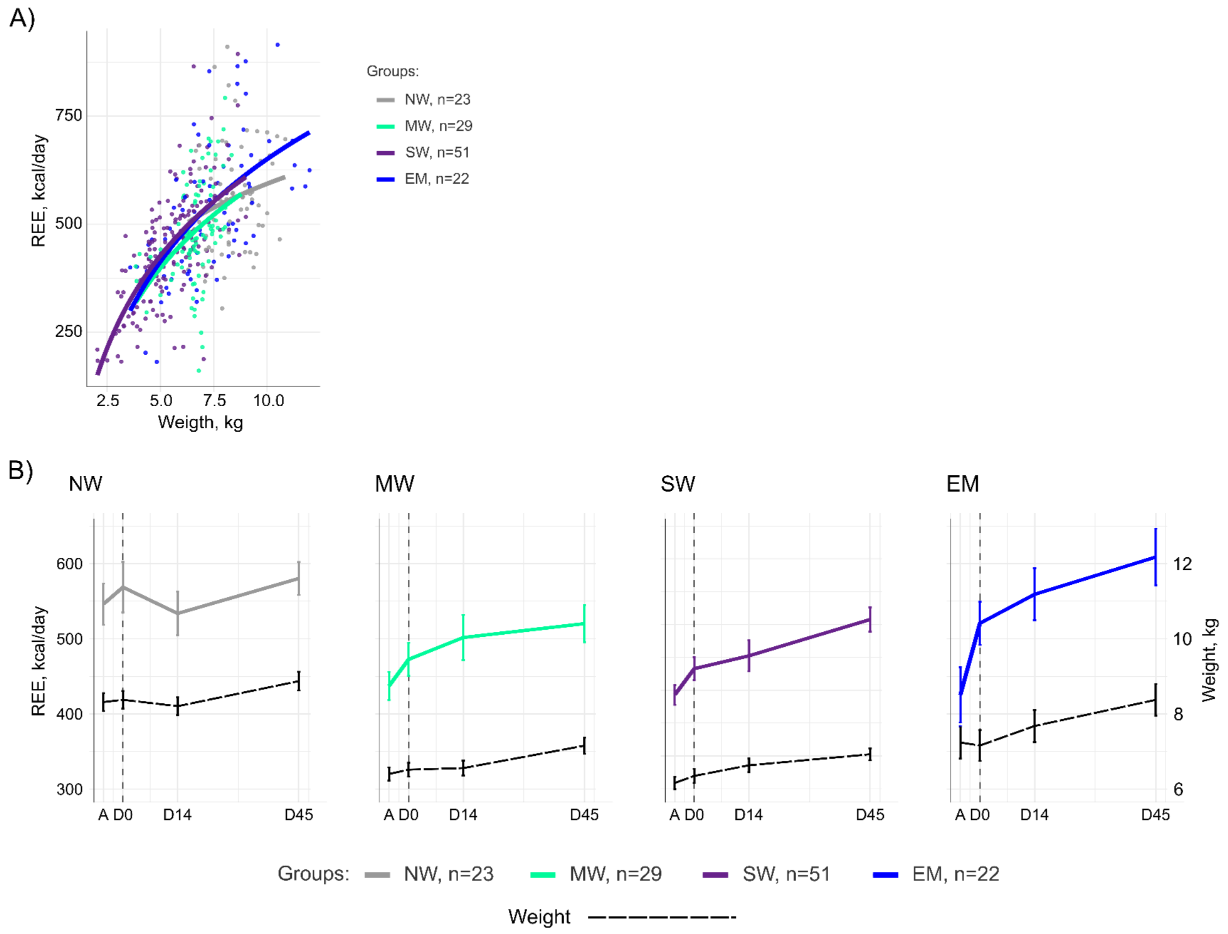

Supplementary Figure S3. Relationship between body weight (kg) and REE (kcal/day) of children represented split by nutritional status as measured with indirect calorimetry. A) Weight in function of REE colored as per legend. B) Dual axis plots, REE trajectory across timepoint colored as per legend (related to left y-axis) in relation to changes in weight (black, right y-axis). NW, no wasting; MW, moderate wasting; SW, severe wasting; EM, edematous malnutrition; REE, resting energy expenditure.

Supplementary Figure S4. Resting energy expenditure (REE, kcal/kg/day) of children with edematous malnutrition at admission (A) and discharge (D0).

REE is corrected by weight at admission (in blue) versus weight at discharge (i.e., after significant loss of edema) (in grey). Lines represent mean group trajectory colored coded as per legend. Error bars indicate standard error of the mean. EM, edematous malnutrition.

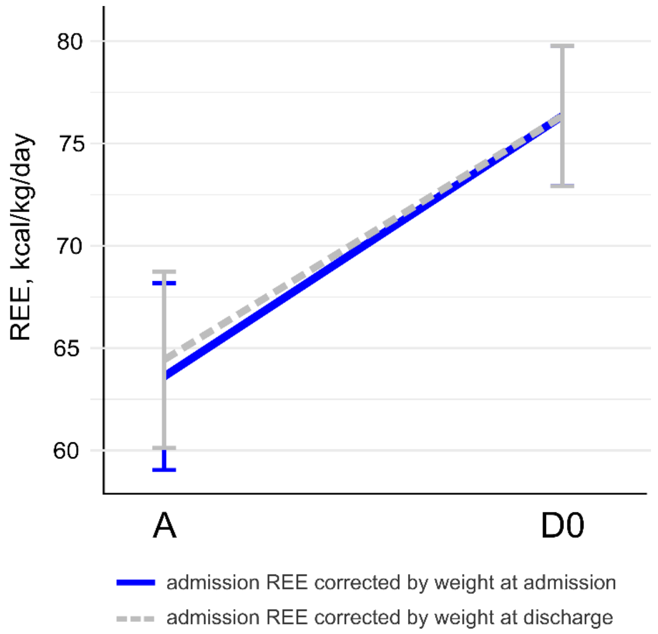

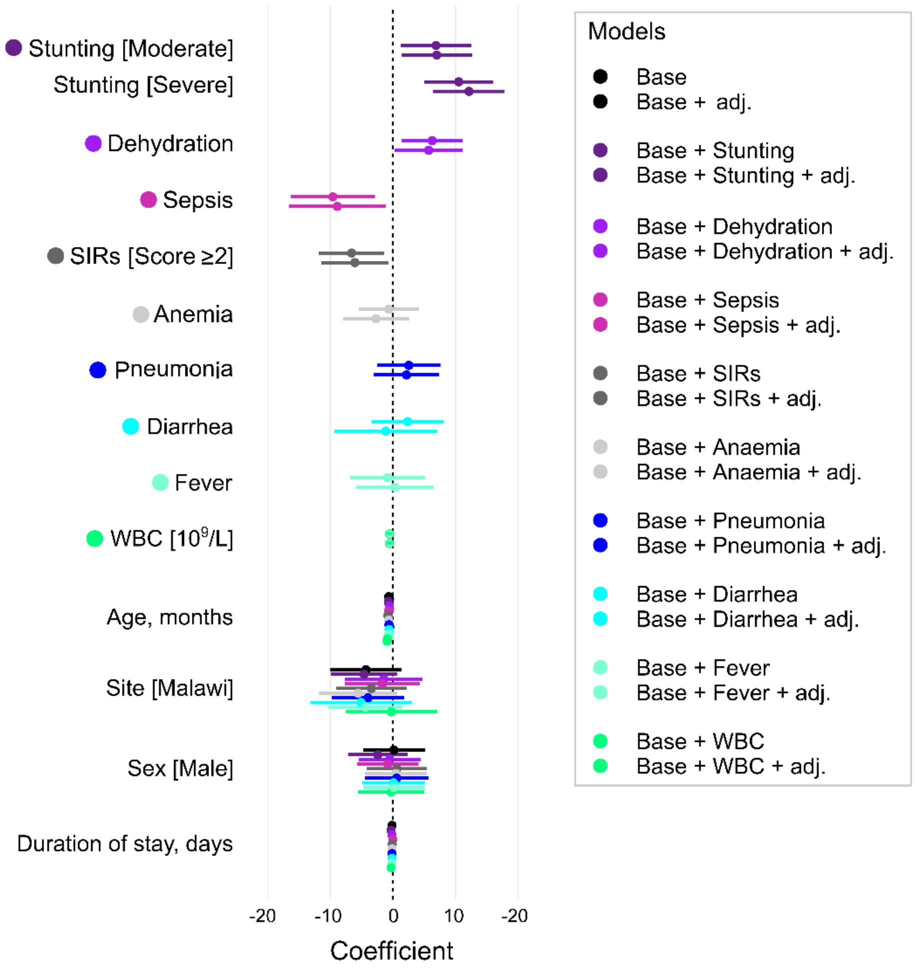

Supplementary Figure S5. Regression coefficients derived from piecewise mixed models testing the association between resting energy expenditure and different clinical diagnoses in children hospitalized with acute illness and of varying nutritional status. These models present coefficients without or with further adjustment (+ adj.) for sex, site and duration of hospital stay. All models include the `Base` model (i.e., M_0: REE ~ time x group + age) with an additional clinical variable as per legend. Piecewise models were fitted with a single knot at discharge and with random intercepts per participant. The confidence intervals of significant variables do not cross zero (i.e., dashed black center line).

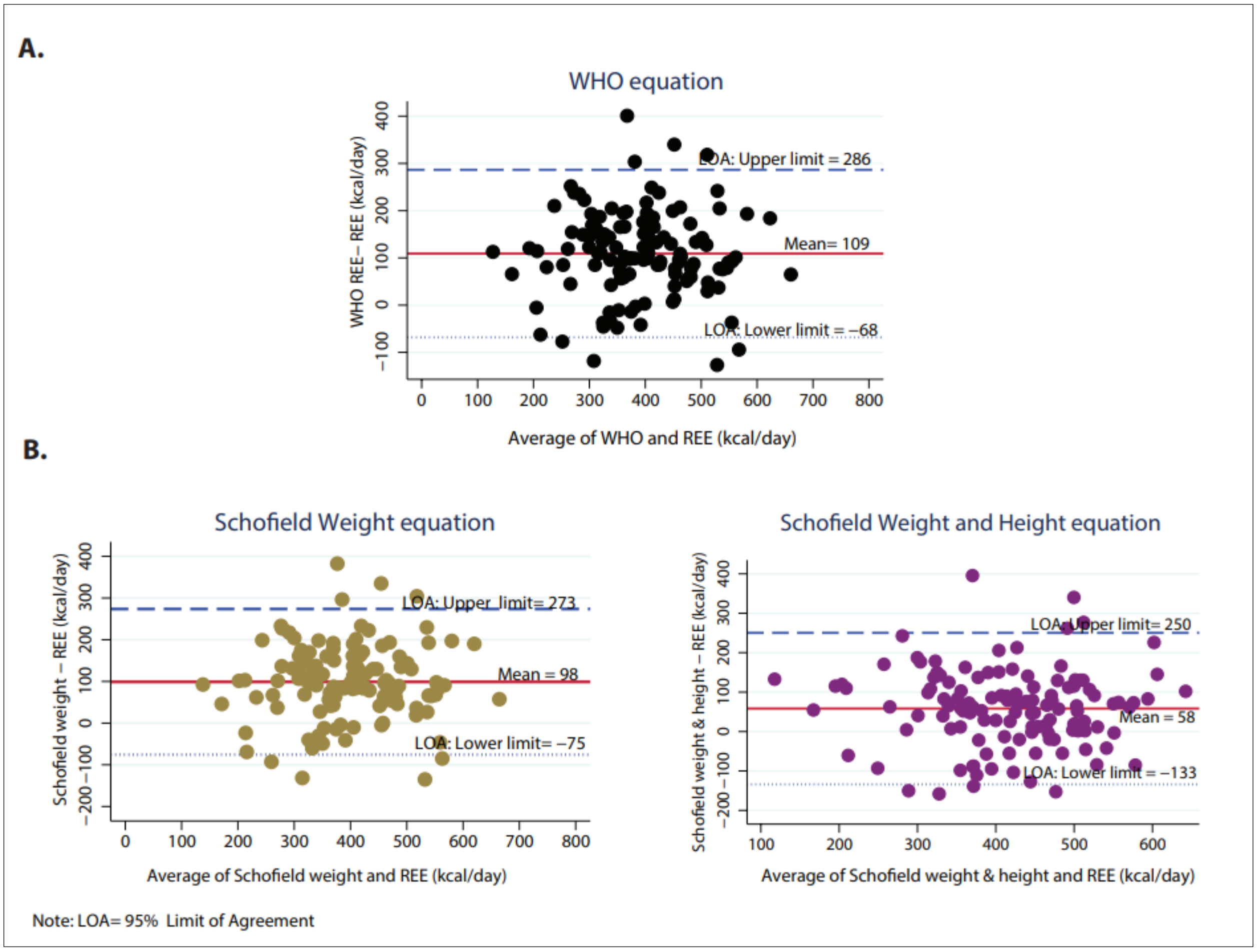

Supplementary Figure S6. Bland-Altman plots comparing absolute differences between measured REE using indirect calorimetry and predicted REE (Y axis), and average of measured and predicted REE (X axis) for the three predictive equations. The horizontal lines representing the mean and the 95% of confidence interval limits for mean. Panel A showing WHO equation, and panel B showing Schofield weight and height-based equations.

STROBE Statement—Checklist of items that should be included in reports of ***cohort studies***

	Item No	Recommendation	Location in the manuscript
**Title and abstract**	1	(*a*) Indicate the study's design with a commonly used term in the title or the abstract	Abstract
		(*b*) Provide in the abstract an informative and balanced summary of what was done and what was found	Abstract
**Introduction**			
Background/rationale	2	Explain the scientific background and rationale for the investigation being reported	Abstract, introduction
Objectives	3	State specific objectives, including any prespecified hypotheses	Abstract, introduction,
**Methods**			
Study design	4	Present key elements of study design early in the paper	Abstract, Methods
Setting	5	Describe the setting, locations, and relevant dates, including periods of recruitment, exposure, follow-up, and data collection	Abstract, Methods
Participants	6	(*a*) Give the eligibility criteria, and the sources and methods of selection of participants. Describe methods of follow-up	Methods
		(*b*) For matched studies, give matching criteria and number of exposed and unexposed	NA
Variables	7	Clearly define all outcomes, exposures, predictors, potential confounders, and effect modifiers. Give diagnostic criteria, if applicable	Methods
Data sources/ measurement	8	For each variable of interest, give sources of data and details of methods of assessment (measurement). Describe comparability of assessment methods if there is more than one group	Methods, Figure 1
Bias	9	Describe any efforts to address potential sources of bias	Methods
Study size	10	Explain how the study size was arrived at	Methods
Quantitative variables	11	Explain how quantitative variables were handled in the analyses. If applicable, describe which groupings were chosen and why	Methods
Statistical methods	12	(*a*) Describe all statistical methods, including those used to control for confounding	Methods

		(*b*) Describe any methods used to examine subgroups and interactions	Methods
		(*c*) Explain how missing data were addressed	Methods
		(*d*) If applicable, explain how loss to follow-up was addressed	NA
		(*e*) Describe any sensitivity analyses	NA
**Results**			
Participants	13	(a) Report numbers of individuals at each stage of study—eg numbers potentially eligible, examined for eligibility, confirmed eligible, included in the study, completing follow-up, and analysed	Result, Figure S1
		(b) Give reasons for non-participation at each stage	Figure S1
		(c) Consider use of a flow diagram	Figure S1
Descriptive data	14	(a) Give characteristics of study participants (eg demographic, clinical, social) and information on exposures and potential confounders	Result, Table 1
		(b) Indicate number of participants with missing data for each variable of interest	NA
		(c) Summarise follow-up time (eg, average and total amount)	Result, Figure 2,
Outcome data	15	Report numbers of outcome events or summary measures over time	Results, Table 2-3, Figure 2-4 Supplementary Tables
Main results	16	(*a*) Give unadjusted estimates and, if applicable, confounder-adjusted estimates and their precision (eg, 95% confidence interval). Make clear which confounders were adjusted for and why they were included	Results, Table 2-3, Supplementary Tables
		(*b*) Report category boundaries when continuous variables were categorized	NA
		(*c*) If relevant, consider translating estimates of relative risk into absolute risk for a meaningful time period	NA
Other analyses	17	Report other analyses done—eg analyses of subgroups and interactions, and sensitivity analyses	NA
**Discussion**			
Key results	18	Summarise key results with reference to study objectives	Discussion
Limitations	19	Discuss limitations of the study, taking into account sources of potential bias or imprecision. Discuss both direction and magnitude of any potential bias	Discussion
Interpretation	20	Give a cautious overall interpretation of results considering objectives, limitations, multiplicity of	Abstract, Discussion

		analyses, results from similar studies, and other relevant evidence	
Generalisability	21	Discuss the generalisability (external validity) of the study results	Discussion
**Other information**			
Funding	22	Give the source of funding and the role of the funders for the present study and, if applicable, for the original study on which the present article is based	Acknowledgments, Funding
